# Associations between Intimate Partner Violence and Termination of Pregnancy: A Systematic Review and Meta-Analysis

**DOI:** 10.1371/journal.pmed.1001581

**Published:** 2014-01-07

**Authors:** Megan Hall, Lucy C. Chappell, Bethany L. Parnell, Paul T. Seed, Susan Bewley

**Affiliations:** Women's Health Academic Centre, King's College London, London, United Kingdom; University of Ottawa, Canada

## Abstract

**Background:**

Intimate partner violence (IPV) and termination of pregnancy (TOP) are global health concerns, but their interaction is undetermined. The aim of this study was to determine whether there is an association between IPV and TOP.

**Methods and Findings:**

A systematic review based on a search of Medline, Embase, PsycINFO, and Ovid Maternity and Infant Care from each database's inception to 21 September 2013 for peer-reviewed articles of any design and language found 74 studies regarding women who had undergone TOP and had experienced at least one domain (physical, sexual, or emotional) of IPV. Prevalence of IPV and association between IPV and TOP were meta-analysed. Sample sizes ranged from eight to 33,385 participants. Worldwide, rates of IPV in the preceding year in women undergoing TOP ranged from 2.5% to 30%. Lifetime prevalence by meta-analysis was shown to be 24.9% (95% CI 19.9% to 30.6%); heterogeneity was high (*I* ^2^>90%), and variation was not explained by study design, quality, or size, or country gross national income per capita. IPV, including history of rape, sexual assault, contraceptive sabotage, and coerced decision-making, was associated with TOP, and with repeat TOPs. By meta-analysis, partner not knowing about the TOP was shown to be significantly associated with IPV (pooled odds ratio 2.97, 95% CI 2.39 to 3.69). Women in violent relationships were more likely to have concealed the TOP from their partner than those who were not. Demographic factors including age, ethnicity, education, marital status, income, employment, and drug and alcohol use showed no strong or consistent mediating effect. Few long-term outcomes were studied. Women welcomed the opportunity to disclose IPV and be offered help. Limitations include study heterogeneity, potential underreporting of both IPV and TOP in primary data sources, and inherent difficulties in validation.

**Conclusions:**

IPV is associated with TOP. Novel public health approaches are required to prevent IPV. TOP services provide an opportune health-based setting to design and test interventions.

*Please see later in the article for the Editors' Summary*

## Abstract 2

Lucy Chappell and colleagues conduct a systematic review and meta analysis to investigate a possible association between intimate partner violence and termination of pregnancy.

*Please see later in the article for the Editors' Summary*

## Editors' Summary

**Background:**

Intimate partner violence (sometimes referred to as domestic violence) is one of the commonest forms of violence against women and is a global health problem. The World Health Organization defines intimate partner violence as any act of physical, psychological, or sexual aggression or any controlling behavior (for example, restriction of access to assistance) perpetrated by the woman's current or past intimate partner. Although men also experience it, intimate partner violence is overwhelmingly experienced by women, particularly when repeated or severe. Studies indicate that the prevalence (the percentage of a population affected by a condition) of intimate partner violence varies widely within and between countries: the prevalence of intimate partner violence among women ranges from 15% in Japan to 71% in Ethiopia, and the lifetime prevalence of rape (forced sex) within intimate relationships ranges from 5.9% to 42% across the world, for example. Overall, a third of women experience intimate partner violence at some time during their lifetimes. The health consequences of such violence include physical injury, depression, suicidal behavior, and gastrointestinal disorders.

**Why Was This Study Done?:**

Intimate partner violence can also lead to gynecological disorders (conditions affecting the female reproductive organs), unwanted pregnancy, premature labour and birth, and sexually transmitted infections. Because violence may begin or intensify during pregnancy, some countries recommend routine questioning about intimate partner violence during antenatal care. However, women seeking termination of pregnancy (induced abortion) are not routinely asked about intimate partner violence. Every year, many women worldwide terminate a pregnancy. Nearly half of these terminations are unsafe, and complications arising from unsafe abortions are responsible for more than 10% of maternal deaths (deaths from pregnancy or childbirth-related complications). It is important to know whether intimate partner violence and termination of pregnancy are associated in order to develop effective strategies to deal with both these global health concerns. Here, the researchers conducted a systematic review and meta-analysis to investigate the associations between intimate partner violence and termination or pregnancy. A systematic review identifies all the research on a given topic using predefined criteria; meta-analysis combines the results of several studies.

**What Did the Researchers Do and Find?:**

The researchers identified 74 studies that provided information about experiences of intimate partner violence among women who had had a termination of pregnancy. Data in these studies indicated that, worldwide, intimate partner violence rates among women undergoing termination ranged from 2.5% to 30% in the preceding year and from 14% to 40% over their lifetime. In the meta-analysis, the lifetime prevalence of intimate partner violence was 24.9% among termination-seeking populations. The identified studies provided evidence that intimate partner violence was associated with termination and with repeat termination. In one study, for example, women presenting for a third termination were more than two and a half times more likely to have a history of physical or sexual violence than women presenting for their first termination. Moreover, according to the meta-analysis, women in violent relationships were three times as likely to conceal a termination from their partner as women in non-violent relationships. Finally, the studies indicated that women undergoing terminations of pregnancy welcomed the opportunity to disclose their experiences of intimate partner violence and to be offered help.

**What Do These Findings Mean?:**

These findings indicate that intimate partner violence is associated with termination of pregnancy and that a woman's partner not knowing about the termination is a risk factor for intimate partner violence among women seeking termination. Overall, the researchers' findings support the concept that violence can lead to pregnancy and to subsequent termination of pregnancy, and that there may be a repetitive cycle of abuse and pregnancy. The accuracy of these findings is limited by heterogeneity (variability) among the included studies, by the likelihood of underreporting of both intimate partner violence and termination in the included studies, and by lack of validation of reports of violence through, for example, police reports. Nevertheless, health-care professionals should consider the possibility that women seeking termination of pregnancy may be experiencing intimate partner violence. In trying to prevent repeat terminations, health-care professionals should be aware that while focusing on preventing conception may reduce the chances of a woman becoming pregnant, she may still be vulnerable to abuse. Finally, given the clear associations between intimate partner violence and termination of pregnancy, the researchers suggest that termination services represent an appropriate setting in which to test interventions designed to reduce intimate partner violence.

**Additional Information:**

Please access these websites via the online version of this summary at http://dx.doi.org/10.1371/journal.pmed.1001581.

- The World Health organization provides detailed information about intimate partner violence and about termination of pregnancy (some information available in several languages)
- MedlinePlus provides links to other resources about intimate partner violence and about termination of pregnancy (in English and Spanish)
- The World Bank has a webpage that discusses the role of the health sector in preventing gender-based violence and a webpage with links to other resources about gender-based violence
- The Gender and Health Research Unit of the South African Medical Research Council provides links to further resources about intimate partner violence (research briefs/policy briefs/fact sheets/research reports)
- DIVERHSE (Domestic & Interpersonal Violence: Effecting Responses in the Health Sector in Europe) is a European forum for health professionals, nongovernmental organizations, policy-makers, and academics to share their expertise and good practice in developing and evaluating interventions to address violence against women and children in a variety of health-care settings
- London School of Hygiene & Tropical Medicine's Gender Violence and Health Centre also has a number of research resources
- The UK National Health Service Choices website provides personal stories of intimate partner violence during pregnancy
- The March of Dimes provides information on identifying intimate partner violence during pregnancy and making a safety plan

## Introduction

Intimate partner violence (IPV) has been defined by the World Health Organization (WHO) as “behaviour within an intimate relationship that causes physical, sexual or psychological harm, including acts of physical aggression, sexual coercion, psychological abuse and controlling behaviours” encompassing both current and past intimate partners [1]. Estimated prevalence varies globally and within countries, and is partly dependent on definition and methodology; lifetime exposure has been found to range from 15% in Japan to 71% in Ethiopia (estimated by WHO multi-country studies), has been estimated at 24% in the UK based on UK Home Office crime statistics [2], and has been estimated to be around 35% (inclusive of stalking) in the US [3]. Rape within intimate relationships has been reported to be common across a number of continents, with lifetime prevalence of forced sex ranging from 5.9% to 42% [4].

Health consequences of IPV are known to include, but not be limited to, increased physical injuries and gastrointestinal, gynaecological, and psychiatric co-morbidities [4]–[7]. Violence may begin or intensify during pregnancy and is associated with adverse obstetric outcomes [8] and maternal death [9],[10]. Increased homicide [5],[11],[12] and suicide are found among individuals experiencing IPV [5],[13].

Randomised trial evidence has shown that training primary care professionals in selective questioning of women about IPV increases disclosure and referral to specialist IPV services [14]. Antenatal routine questioning for IPV is recommended in both the US and UK [15],[16], despite uncertainty over the harms and benefits of universal questioning and subsequent intervention [17]. Ongoing pregnancy is considered to be a time of increased risk of IPV, yet women seeking termination of pregnancy (TOP) are not such a focus of attention [18]. An evidence-based understanding of the association between IPV and TOP would directly inform the development of strategies for effective interventions for IPV. To our knowledge there has been no previous systematic review of the literature.

The aim of this study was to determine whether there is an association between IPV and TOP.

## Methods

### Selection Criteria

Studies were considered eligible for inclusion if they (1) included women who were seeking or had undergone a TOP and studied at least one aspect of IPV in this group; (2) were a randomised control trial, case-control study, cohort study, cross-sectional analysis, experimental study, or secondary study with data of interest; and (3) were peer reviewed. Studies focusing on violence by individuals other than current or former intimate partners were excluded. No restrictions were placed on the setting, time, or language of the studies. Quantitative data were not necessary for inclusion.

### Search Strategy

The population of interest was women seeking or having undergone a TOP in any setting; the exposure was the presence or absence of IPV; the control group, where reported, was a separate cohort of comparable individuals (e.g., pregnant women not seeking TOP, pregnant women not reporting IPV, or women attending a gynaecology clinic); the statistic of interest was the association between IPV and TOP. We included articles where the relative timing of IPV and TOP could not be delineated. The search strategy was devised using a combination of Medical Subject Headings (MeSH terms) and free text terms with synonyms (see Table 1). Searches were carried out in Medline (1946–21 September 2013), Embase (1980–21 September 2013), PsycINFO (1806–21 September 2013), and Ovid Maternity and Infant Care (1971–21 September 2013) from the earliest possible date until 21 September 2013. In addition, a search of Web of Science and hand searches of reference lists of all included articles were carried out. Nine authors were contacted regarding results, and they identified further articles. There was no restriction on language. If multiple articles based on the same study were identified, duplication was avoided by only using the data reported for different sub-groups.

**Table 1:** Search strategy.

Database	Dates Searched	Search Terms 1	Search Terms 2	Search Terms 3	Limitations
Ovid Medline	1946–21 September 2013	Abortion, induced/OR abortion, therapeutic/OR induce* abortion*.mp OR therapeutic abortion*.mp OR medical abortion*.mp OR termination of pregnancy.mp	Domestic violence/OR spouse abuse/OR battered women/OR domestic violence.mp OR spouse abuse.mp OR domestic abuse.mp OR battered women.mp OR battered female.mp OR intimate partner violence.mp OR partner abuse.mp OR wife beating.mp OR battering.mp	1 AND 2	LIMIT 3: Human
OVID Embase	1980–21 September 2013	As for Ovid Medline	As for Ovid Medline	1 AND 2	LIMIT 3: Human
OVID PsycINFO	1806–21 September 2013	Abortion, induced/OR induce* abortion*.mp OR therapeutic abortion*.mp OR medical abortion*.mp OR termination of pregnancy.mp	Domestic violence/OR partner abuse/OR battered females/OR domestic violence.mp OR spouse abuse.mp OR domestic abuse.mp OR battered women.mp OR battered female.mp OR intimate partner violence.mp OR partner abuse.mp OR wife beating.mp OR battering.mp	1 AND 2	LIMIT 3: Human
OVID Maternity and Infant Care	1971–21 September 2013	Induce* abortion*.mp OR therapeutic abortion*.mp OR medical abortion*.mp OR termination of pregnancy.mp	Domestic violence.mp OR spouse abuse.mp OR domestic abuse.mp OR battered women.mp OR battered female.mp OR intimate partner violence.mp OR partner abuse.mp OR wife beating.mp OR battering.mp	1 AND 2	

### Data Extraction

All titles were independently screened by two authors (M. H. and S. B.). If either considered a title relevant, both reviewers independently screened the abstract. All articles were included for full-text assessment if either author considered the abstract relevant or there was uncertainty. Full-text assessment to determine inclusion was independently carried out by two authors (M. H. and S. B.). Any disagreements were discussed, and any study whose inclusion remained ambivalent was referred to a third author (L. C. C.).

A standard form was devised (see Table S1) prior to data extraction and quality scoring. Data were extracted on study type; population; setting, country, and region; demographic and health factors; intervention; comparator population; definition of IPV; screening tools used; and incidence and prevalence of IPV among general populations and in relation to TOP. Data from the articles were independently extracted by two authors (M. H. and B. L. P.). Results addressing violence by individuals other than current or previous intimate partners were excluded. Any uncertainties were discussed, and referred to a third author (L. C. C.) if necessary. Data extraction and quality scoring of articles published in languages other than English (*n* = 3) were undertaken by one author (M. H.) and the translator.

### Quality Appraisal

Quality appraisal of quantitative and qualitative studies was carried out using Critical Appraisal Skills Programme (CASP) scales [19] as modified by Oram and colleagues [20] (see Tables S2 and S3), consisting of 15 and ten criteria, respectively, each of which could be scored between zero and two (maximum scores 30 and 20). Two authors (M. H. and B. L. P.) carried out full quality appraisal of all articles. Any disagreements were discussed, and referred to a third author (L. C. C.) if necessary. Quantitative CASP scores of ≥25, ≥20, ≥15, and ≤14 and qualitative CASP scores of ≥17, ≥14, ≥12, and ≤11 were considered high, medium, low, and very low quality, respectively.

### Meta-Analysis and Regression

The prevalences of IPV (as percentages) were converted to log odds prior to combining using the DerSimonian and Laird random effects method of meta-analysis [21]. Resulting estimates and confidence intervals were reconverted to percentages prior to display. Forest plots show actual percentages on the log-odds scale and are displayed sub-grouped by gross national income (GNI) per capita, as previous reports (e.g., from WHO) have chosen similar economic groupings [22]. Comparisons between groups used odds ratios (ORs). Estimated heterogeneity (*I* ^2^) is displayed for all group and sub-group analyses. We investigated the possibility of a sub-group of recent or high-quality studies with consistent methods and consistent results that could be used to give generally applicable results.

Meta-analysis regression is a meta-analysis technique developed specifically to explain large and unexplained differences in results between studies (also known as heterogeneity). The method used assumes that the differences are in part random and in part explainable [23].

Potential sources of heterogeneity were investigated using meta-analysis regression: country's GNI per capita (in intervals of INT$10,000), study quality (measured by CASP score), date of study (decade), study design (cross-sectional, cohort, or case-control), setting (urban versus regional versus national), and study size (total number of participants as a continuous variable). Egger's test was performed in order to assess potential publication bias [24].

The review was performed according to protocol (Text S1) and in line with PRISMA guidelines (Table S4).

## Results

### Study Selection Process

**Figure 1:**
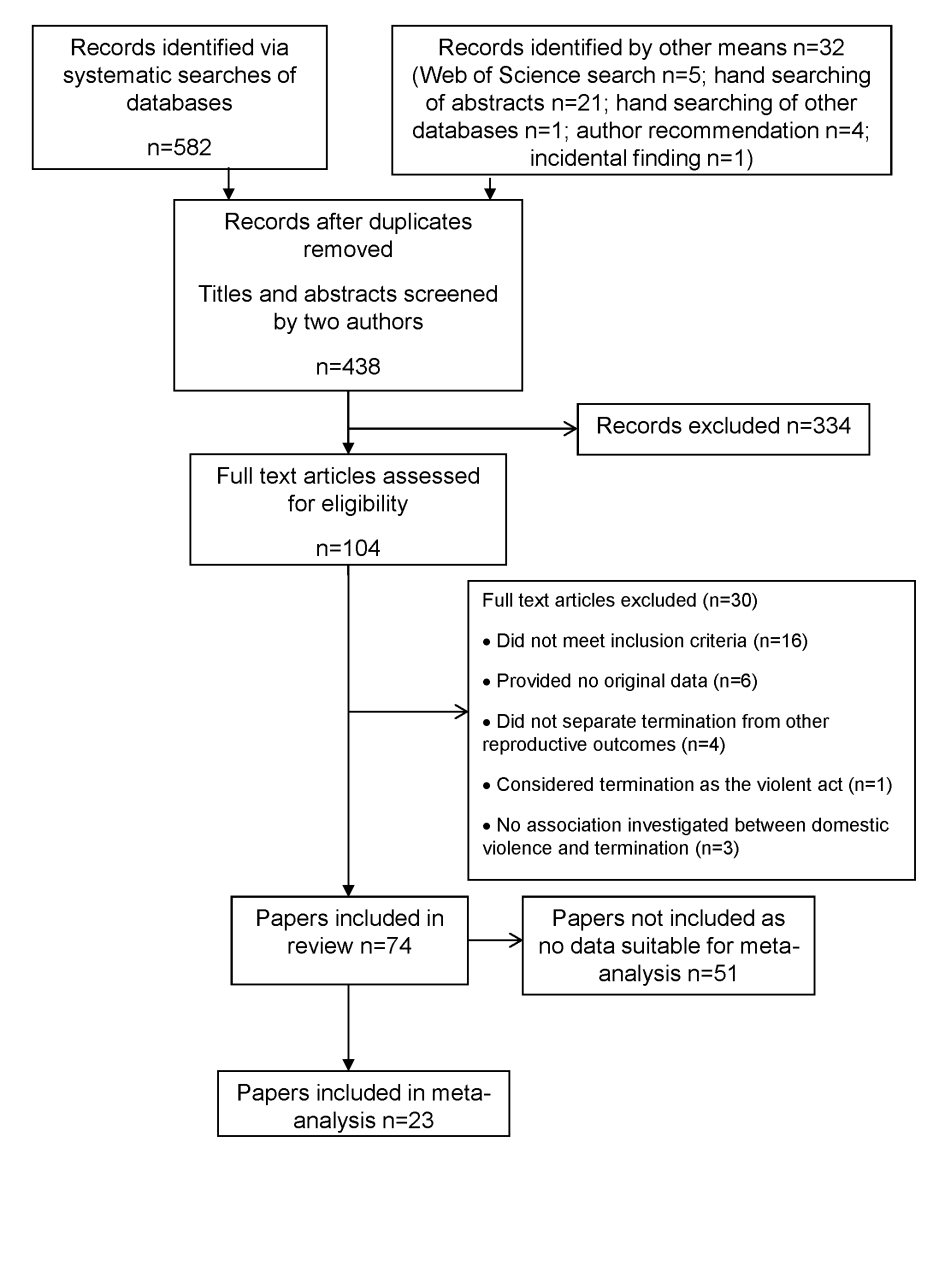
PRISMA flow diagram.

The study selection process is shown in Figure 1. Of a total of 438 articles identified for screening after removal of duplicates, 104 were considered eligible for full-text screening, and 74 were included in the review [25]–[98]. Tables 2–9 detail the studies, grouped into tables by design—and additionally by continent for cross-sectional studies—and listed within tables by reverse chronology.

**Table 2:** Characteristics of included cohort studies.

Study	Population; Country	Exposure	Outcome	CASP Score (/30)
Fergusson et al. 2007 [25]	492 women participating in a 25-y longitudinal study of a New Zealand birth cohort; New Zealand.	Pregnancy ending in TOP before 21 y of age.	Outcome was social and economic outcomes for women aged 21–25 y. Women who had become pregnant and not had a TOP had consistently poorer outcomes (reduced educational achievement, lower income, higher welfare dependence, poorer partner relationships—including exposure to partner violence). There was a significant tendency for pregnancy without TOP to be associated with a higher rate of exposure to partner violence (*p*<0.01).	22
Taft and Watson 2007 [26]	Cohorts of Australian women aged 18–23 y taking part in a long-term longitudinal study (*n* = 14,779); Australia.	1-y history of physical and/or sexual violence. Lifetime history of violent relationship.	Women who reported ever experiencing partner violence had OR of 2.65 (95% CI 1.96 to 3.60) for TOP compared to non-abused women. Women who reported partner and recent physical or sexual violence had even higher odds (OR = 3.52, 95% CI 2.14 to 5.81).	19
Stenson et al. 2001 [27]	All women (*n* = 1,038) registered for antenatal care in Uppsala, and who gave birth there, between September 1997 and February 1998; Sweden.	Lifetime, pregnancy, and year prior to pregnancy histories of physical and/or sexual abuse. Fear of partner.	Women who reported abuse had undergone more TOPs than those who did not (*p*<0.001). 30 of 837 women who did not report IPV reported multiple TOPs, as opposed to 7 of 14 women reporting IPV (*p*<0.001).	24

**Table 3:** Characteristics of included case-control studies.

Study	Population; Country	Exposure	Comparison	Outcome	CASP Score (/30)
Gee et al. 2009 [28]	1,463 women aged 18 y and over presenting for TOP at a Planned Parenthood Center; US.	Lifetime and 12-mo history of physical and sexual IPV. Partner willingness to use, and having control of, contraception.	Women presenting to general gynaecology clinic.	21% women reported history of IPV. By a multivariate model, women who had experienced IPV were more likely to report lack of birth control use due to partner unwillingness to use birth control, prevention of access to birth control, or the partner's desire for the woman to become pregnant. Women who reported IPV were also significantly more likely to have reported going without birth control in the past 4 mo (70.9% of 285 women reporting IPV, compared to 64.5% of 698 women not reporting IPV). Numbers of TOPs significantly associated with IPV—with each additional TOP there is a 16% increased odds that woman has a positive IPV history.	23
Romito et al. 2009 [29]	445 women undergoing TOP at a hospital over a certain period of time; Italy.	12-mo history of physical, psychological, or sexual violence. Current physical, psychological, or sexual violence.	Women having live birth in the same hospital.	Physical and psychological violence were more prevalent among the TOP-seeking population than among the live birth group (4.6% versus 0.9% and 11.0% versus 2.5%, respectively, *p*<0.001 in both cases). There was no significant difference in rates of sexual violence among the two groups (1.8% versus 0.5%, *p* = 0.056).	25
Bourassa and Berube 2007 [30]	All (350) women who presented for voluntary TOP at a family planning clinic in Quebec; Canada.	Lifetime abuse by partner. Past year psychological, physical, and/or sexual abuse by partner. Physical abuse during pregnancy by partner.	Women presenting to a perinatal nurse as part of ongoing pregnancy care.	Women presenting for TOP were at higher risk than the control group for IPV. Prevalence ratios: 1.41 lifetime abuse; 2.75 past year IPV; 3.88 physical/sexual IPV past year. Single women were more likely to report IPV (*p*<0.0001).	25
Lipsky et al. 2005 [31]	Women 16–41 y who reported IPV to Seattle police department between 1995 and 1998, and who subsequently filed a singleton live birth or fetal death with the State of Washington that indicated that they were pregnant within the timeframe of the incident of violence (*n* = 389); US.	IPV reported to the police.	Women who filed a singleton live birth or fetal death with Washington State 1995–1998, but who had no history of violence reported to the police (*n* = 3,090).	Population of interest rate of TOP = 34%; control group rate of TOP = 24% (significant difference).	17
Helweg-Larsen and Kruse 2003 [32]	1,815 women aged 15–49 y who presented to hospital in 1995 with injuries resulting from IPV; Denmark.	Physical injury from IPV resulting in hospital attendance.	Women aged 15–49 y who presented to hospital in 1995 for reasons other than IPV-related injuries.	Women who had presented for violence-related injuries were more likely to have a TOP within the following year. Abused women aged 20–29 y were more likely to have a TOP at any stage in the follow-up than their non-abused counterparts.	22
Leung et al. 2002 [33]	245 patients requesting TOP at Hong Kong hospital; China.	Lifetime, past year, and current pregnancy history of physical violence. Lifetime history of emotional violence. Past year history of sexual violence. Living in fear. Whether or not violence has affected decision to have TOP.	General gynaecology patients (*n* = 256).	Lifetime history of physical, emotional, and sexual abuse was significantly higher among the TOP-seeking population than the control group. The same was also found to be true of the past year history of physical or sexual violence, and sexual violence alone (all *p*<0.001). Past year history of physical violence alone was also significantly higher in the TOP group (*p*<0.01). There was no significant difference in numbers of women living in fear among the two groups. Boyfriends were more likely to carry out physical violence than husbands, although the two groups were equally likely to perpetrate sexual violence.	24
Yimin et al. 2002 [34]	1,137 unmarried women, under the age of 22 y, presenting for TOP who reported a history of sexual coercion; China.	Beaten or abused by partner.	1,246 women presenting for TOP who did not report a history of sexual coercion.	Women who reported sexual coercion were also more likely to report being abused or beaten (*p*<0.01 in both cases).	18
Yimin et al. 2001 [35]	667 women presenting for TOP who reported a history of sexual coercion; China.	Abused or experienced battery at hands of partner.	726 women presenting for TOP who did not report a history of sexual coercion.	Women who reported sexual coercion were also more likely to report abuse or battery (*p*<0.01 for both).	15

**Table 4:** Characteristics of included cross-sectional studies from the Americas.

Study	Population; Country	Exposure	Outcome	CASP Score (/30)
Jones et al. 2013 [36]	9,493 women seeking second trimester TOP; US.	Physically hurt or forced to participate in sexual activities by father of current pregnancy.	13.7% of women who required a TOP at ≥12 weeks' gestation reported violence. At ≥16 weeks' gestation, this percentage rose to 39.1% (OR 1.23, 95% CI 0.79 to 1.91).	19
Ely et al. 2011 [37]	120 unmarried TOP patients aged 14–21 y; US.	Abuse at hands of co-conceiving partner. CADRI	4% of respondents reported experiencing abuse at hands of co-conceiving partner. Mean CADRI score: 115.9 (standard deviation 26.6, *n* = 96).	18
Ely and Otis 2011 [38]	188 women aged 18–46 y seeking TOP at a clinic in southeast US; US.	Emotional, physical, and/or sexual abuse victimisation in the past 30 d.	14.2% reported emotional abuse, 6.4% physical abuse, 3.9% sexual abuse (numbers include overlap). Women who reported a history of previous TOPs were more likely to also report that they had been abused by the father of their pregnancy (*χ* ^2^ = 5.20, *p*≤0.05, Cramer's *V* = 0.171). Personal stress, depression, reduced self-esteem, and increased sexual discord were all found to be significantly associated with IPV among women seeking TOP. 1.4% of 138 women not reporting IPV reported a partner refusing to use a condom, as compared to 52% of 25 women reporting IPV.	17
Jones et al. 2011 [39]	9,493 women presenting for TOP. Required ability to read any of English, Spanish, or Portuguese; US.	IPV perpetrated by co-conceiving partner. Co-conceiving partner's involvement in TOP.	6%, 3%, and 7% respondents described physical, sexual, and emotional violence, respectively, at hands of co-conceiving partner. Exposure to IPV reduces the likelihood of the woman believing her partner to know about the TOP (OR 0.28, 95% CI 0.21 to 0.37).	22
Jones and Finer 2012 [40]	9,493 women seeking TOP; US.	Physical or sexual violence perpetrated by father of pregnancy.	654/9,493 women reported IPV, with 549 reports of physical and 243 reports of sexual violence.	24
Roth et al. 2011 [41]	1,060 pregnant women who were ≤63 d gestation and were recruited for a medical abortion trial; US.	Lifetime and current pregnancy history of physical and sexual violence.	21.6% of women reported experiencing IPV. These women were significantly more likely to have a history of prior TOPs (*p* = 0.02), and were also more likely to have discrepancy between GA calculated from last menstrual period and ultrasound GA (*p*<0.001). No significant association between IPV and age was noted, with women reporting IPV having an average age of 26.0±5.5 y, as compared to 25.9±5.8 y among women not reporting IPV. Women reporting IPV were more likely to be single (OR 3.0, 95% CI 1.7 to 5.3) or divorced, separated, or widowed (OR 3.0, 95% CI 1.9 to 7.7) than married.	24
Steinberg and Finer 2011 [42]	2,070 women aged 15–54 y who participated in the National Comorbidity Survey; US.	Physical violence perpetrated by an intimate partner.	30.8% of women reporting one TOP reported IPV; 24.3% of women reporting two TOPs reported IPV; 40.7% of women reporting three TOPs reported IPV.	19
Saftlas et al. 2010 [43]	986 women resident in Iowa and ≥18 y presenting for TOP, with proficiency in English and/or Spanish; US.	12-mo history of physical and/or sexual abuse, and of battering (where battering was defined as “chronic, nonphysical abuse characterised by controlling behaviours and abuse of powers”).	9.9% and 2.5% of participants reported physical and sexual IPV, respectively. 8.4% of women reported battering, with 58.3% of this group reporting battering alone. Women not reporting being in a relationship at the time of recruitment to study reported the highest rates of physical or sexual IPV (16.0%).	22
Silverman et al. 2010 [44]	1,318 English-, Spanish-, or Portuguese-speaking men aged 18–25 y who reported having had sex at any stage in their life. Recruited from community health centres in Boston; US.	Lifetime history of physical and/or sexual violence.	31.9% of participants reported perpetrating physical or sexual violence against a female partner. TOP involvement was more common among men who reported IPV than those who did not (48.9% versus 25.9%; ARR 1.79, 95% CI 1.54 to 2.06). Men reporting IPV perpetration were also more likely to be involved in ≥2 TOPs (ARR 3.39, 95% CI 2.06–5.56).	21
Thiel de Bocanegra et al. 2010 [45]	Women living in IPV shelters in the San Francisco area, who were ≥18 y old, and had been in a violent heterosexual relationship for ≥3 mo prior to entering the shelter (*n* = 53); US.	Birth control sabotage, partner unwillingness to use condoms, forced sex, partner infidelity, and unintended pregnancy.	21/53 [40%] women stated that their partner had told them not to use birth control, with 10 of these women being prevented from obtaining it. 11 women concealed the use of birth control from their partner, and one the use of emergency contraceptive. Two-thirds of women reported being forced to have sex by their partner. A total of 68 unintended pregnancies were reported, with 17 ending in TOP. Women reported both being prevented from obtaining, and being forced to have, TOP.	13
Coleman et al. 2009 [46]	18–61-y-old, non-institutionalised residents of Chicago. Sufficient levels of English or Spanish required for completion of survey. Sexually active with at least one partner within the past 12 mo (906 women, 658 men); US.	Physical IPV within current relationship. Conflict within current relationship.	Women who reported TOP in current relationship had higher violence scores (2.50) compared to those with no history of TOP (1.93) or TOP prior to current partnership (1.86), *p*<0.05 for both adjusted comparisons.	21
Ely et al. 2009 [47]	120 unmarried TOP patients aged 14–21 y; US.	IPV perpetration and/or victimisation in relationship with co-conceiving partner.	Average dating violence score 115.28 (average level). Lowest score: 70; highest score: 224. IPV and TOP were significantly associated with increased stress, aggression, and suicidal ideation among participants, but not depression or reduced self-esteem.	20
Prager et al. 2007 [48]	Consecutive sample of 398 women who received TOP at urban hospital, excluding women seeking TOP for fetal anomaly; US.	IPV and sexual abuse—not specified.	No significant difference was found between rate of violence among women undergoing first TOP and those undergoing repeat TOP (*p* = 0.898).	13
Kazi et al. 2008 [49]	286 women who volunteered for TOP, contraceptive, and other gynaecological research studies; US.	Presence of physical or sexual violence historically, and over the past 2 mo, or during pregnancy (if involved in TOP trial).	No significant difference between any of the groups reporting either historical or recent abuse was noted (*p* = 0.44 and *p* = 0.24, respectively).	22
Finer et al. 2005 [50]	1,209 women seeking TOP from 11 large providers of the service; US.	Husband or partner abusive towards woman or her children. Husband or partner wants woman to have TOP.	3% of women reported having an abusive husband or partner as a reason for having a TOP. 24% of women stated that their reason for having a TOP was that it was what their partner wanted.	18
Fisher et al. 2005 [51]	1,143 women presenting at a regional TOP provider in Ontario; Canada.	Lifetime history of physical abuse by a male partner. History of sexual abuse or coercion.	26.4% of women reported significant conflict with the father of their pregnancy; 19.5% reported physical abuse from at least one male partner; 27% reported past history of sexual violence at any stage of life. Women undergoing repeat TOPs were more likely than those seeking a first TOP to report physical abuse by a male partner, sexual abuse, or sexual violence (*p*<0.001). They were also more likely to report significant conflict with the man involved in current pregnancy (*p*<0.01).	24
Hathaway et al. 2005 [52]	38 women participating in a hospital-based IPV programme; US.	Limitation of reproductive autonomy by male partner.	Seven participants described a partner attempting to force them into TOP [18.4%]. Two of these women underwent TOP [5.3%].	13
Raj et al. 2005 [53]	208 South Asian women in heterosexual relationship living in Boston, MA; US	Physical or sexual abuse, or injury perpetrated by current partner.	Unwanted pregnancy is more likely in the abused population (OR 3.39, 96% CI 1.33 to 8.66). Within abused group there were also descriptions of forced or coerced TOP.	22
Woo et al. 2005 [54]	All English- and/or Spanish-speaking patients seeking TOP at a single clinic in Texas (*n* = 818); US.	Lifetime emotional violence. Lifetime, past year, and pregnancy physical violence. Past year sexual violence. Fear of someone.	13.8% of respondents stated a significant abuse history, and 2.8% reported abuse within the current pregnancy. 17.2% of respondents did not disclose their TOP to their partner. 20.9% of this group stated that this was because the partner would oppose the TOP; 7.9% stated disclosure would result in physical harm. Women who had a history of abuse were less likely to tell their partner about the TOP than those without (*p* = 0.001).	26
Janssen et al. 2003 [55]	4,750 women delivering at >20 weeks' gestation at a hospital in British Columbia; Canada.	Physically abused during current pregnancy; fear of partner during current pregnancy.	Women reporting IPV are more likely to have a past medical history of TOP (*p*<0.03).	18
Winn et al. 2003 [56]	205 patients attending postnatal follow-up in Washington State; US.	Self-disclosed current or past physical or sexual abuse on medical records.	History of abuse associated with TOP (*r* = 0.38, *p*<0.000).	15
Wiebe and Janssen 2001 [57]	254 women attending an abortion clinic in British Columbia; Canada.	Recent IPV.	15% reported IPV within the past 12 mo, with 8.3% reporting physical abuse, 7.1% reporting sexual abuse, and 8.3% stating they were afraid of their partner. No significant association between IPV disclosure and age was noted, with average age of all participants 28.0±6.5 y, and of women reporting IPV 28.0±7.0 y.	16
Letourneau et al. 1999 [58]	191 women attending a general gynaecology clinic (students not included); US.	Lifetime history of physical or emotional abuse perpetrated by intimate partner or someone close to patient. Lifetime history of being forced to have sex.	Victims of violence were more likely to report a history of TOP than those without violence in history (*p*<0.05). Of women reporting violence, 50% had undergone a TOP (20/40).	16
Glander et al. 1998 [59]	486 women aged 18 y or over seeking TOP and reporting history of IPV; US.	Lifetime, recent, and current pregnancy physical violence. Forced sex in relation to conception of current pregnancy and first intercourse.	39.5% respondents identified themselves as having a history of IPV. Women reporting IPV history significantly more likely not to have told their partner about their pregnancy than control group (*p* = 0.02). They were also more likely not to involve the partner in the decision to have a TOP (*p*<0.01).	21
Evins and Chescheir 1996 [60]	51 women self-referring for TOP; US.	Lifetime physical abuse. Past year physical abuse. Abuse during pregnancy. Sexual abuse within past year.	11/51 [22%] women described past year history of IPV. 100% of women battered during ongoing pregnancy were also battered prior to pregnancy. Among 16 women reporting IPV there was a total of 6 previous TOPs, as compared to 10 previous TOPs among 29 women not reporting IPV.	16
Holmes et al. 1996 [61]	4,008 female residents of the US, aged ≥18 y at time of first study; US	Lifetime prevalence of rape; prevalence of rape-related pregnancy; outcomes of rape-related pregnancy.	29.4% of rapes disclosed were perpetrated by a boyfriend and 17% by a husband. Rape-related pregnancy was ended with TOP in 50% of reported cases.	21
Torres and Forrest 1988 [62]	Patients at major providers of TOP in the US. 1,900 patients were included in total, 420 of whom were at ≥16 wk gestation; US.	Reasons for choosing to terminate pregnancy. In particular: fear of telling partner about TOP; feeling pressurised into having TOP by someone close; husband or partner mistreats participant or her children.	1% of women stated that their primary reason for having a TOP is that their partner/husband wanted them to, and 6% stated that their primary reason is that their partner/husband mistreats either them or their children. Fear of telling partner of a pregnancy and/or feeling pressure to have a TOP were both cited as reasons for delaying decision to have TOP. Fear of telling partner (or parents) about pregnancy was also stated as a reason for late TOP, as was being pressurised into not having TOP.	13
Borins and Forsythe 1985 [63]	100 patients attending a women's psychiatry clinic in Toronto; Canada.	Physical and/or sexual abuse as an adult or child.	Physical and/or sexual abuse significantly correlated with TOP: *χ* ^2^ = 10.14, df = 1, *p*<0.001.	15
Diniz et al. 2011 [64]	147 women seeking TOP; Brazil.	Definition not stated.	88% of women reported lifetime history of IPV, with 47% experiencing IPV in the current pregnancy.	13

Percentages in brackets are calculated percentages not reported in the original studies.

ARR, adjusted risk ratio; CADRI, Conflict in Adolescent Dating Relationships Inventory; GA, gestational age.

**Table 5:** Characteristics of included cross-sectional studies from Europe.

Study	Population; Country	Exposure	Outcome	CASP Score (/30)
Laanpere et al. 2013 [65]	2,735 women aged 15–44 y participating in a national household survey; Estonia.	Physical or sexual violence encountered in the past 12 mo, perpetrated by current of former partner.	Among women who reported IPV, 150/362 reported at least one TOP, compared to 604/1,604 women not reporting IPV. Women reporting IPV were more likely to report repeat TOP: adjusted OR 1.72 (95% CI 1.24 to 2.37).	23
Makenzius et al. 2012 [66]	590 men whose partners underwent a TOP at a particular clinic; Sweden	Physical, sexual, and psychological violence perpetrated against the male participants in the past 12 mo.	Among the men whose partners were seeking their first TOP, violence was reported in 24/402 [6%] cases; of those women seeking a second or greater TOP, 23/188 [12%] reported violence, *p* = 0.01.	18
Johnson et al. 2007 [67]	920 women attending a gynaecology outpatient clinic in Hull, England; UK.	Lifetime history of emotional abuse.	Emotional abuse more prevalent among women seeking TOP than among women seeking gynaecological care for other reasons (*p*<0.001, *χ* ^2^ = 17.9).	22
John et al. 2004 [68]	825 women attending a general gynaecology clinic in Hull; UK.	Past year physical violence, forced sexual activity, and fear.	Among women presenting for TOP, 24/86 reported IPV (28%). Among women reporting IPV, 24/171 reported history of IPV (14%). By *χ* ^2^ test this was not significant.	18
Keeling et al. 2004 [69]	All women (312) attending pregnancy counselling clinic in northwest England over 7-mo period. Only women who intended to have TOP were included; UK.	Lifetime and past year history of physical, sexual, and emotional abuse. Current physical abuse. Living in fear.	35.1% (95% CI 29.8 to 40.4) of participants disclosed lifetime physical or emotional abuse. 24.5% of this group were still with perpetrator at time of TOP. 44% of this group described weapon- or non-weapon-related injury to head, and 8.8% described injury to genitals. Prevalence of physical abuse within the past 12 mo was 19.5% (95% CI 14.9 to 24.0); 39.5% of this group reported still being with the perpetrator. Current (past fortnight) abuse was reported by five women. 3.7% (95% CI 1.5 to 5.9) of women reported forced sex within the past 12 mo. 55% of this group thought their pregnancy to be related to this event. 54.6% of these events were perpetrated by a current or former partner or husband. 6.6% of women reported living in fear: 90% of this group had a lifetime history of violence, 45% a past year history.	21
Zsuzsa et al. 2004 [70]	6,980 women participating in a cross-sectional health check of residents aged 18 y or over; Hungary.	Physical violence perpetrated by partner, parents, or relative. In particular, physical violence within past year. Currently living in fear. Stress in marriage.	15.5% of women who reported a TOP also reported physical abuse perpetrated by partner, in contrast to 6.7% of women who did not report a TOP (*p*<0.001, OR 2.529, 95% CI 2.112 to 3.027). Likewise, physical abuse from relatives (*p* = <0.001, OR 1.630, 95% CI 1.370 to 1.940), fear of someone (*p*<0.001, OR 2.082, 95% CI 1.547 to 2.801), and physical abuse in the past year (*p*<0.008, OR 1.675, 95% CI 1.181 to 2.374) were all more common among women reporting TOP.	25
Hedin and Janson 2000 [71]	207 Swedish-born women, with Swedish-born partners, attending an antenatal clinic; Sweden.	Physical and/or sexual violence perpetrated by partner during the current pregnancy.	Of 23 women who reported abuse in the current pregnancy, 10 reported a previous TOP [43%], as compared to 46/184 [25%] women not reporting abuse.	17

Percentages in brackets are calculated percentages not reported in the original studies.

**Table 6:** Characteristics of included cross-sectional studies from Africa.

Study	Population; Country	Exposure	Outcome	CASP Score (/30)
Pallitto et al. 2013 [72]	Ever-partnered women selected for participation in the WHO Multi-Country Study on Women's Health and Domestic Violence against Women (*n* = 17,518); Bangladesh, Brazil, Ethiopia, Japan, Namibia, Peru, Samoa, Serbia, Montenegro, Thailand, United Republic of Tanzania.	Lifetime history of physical or sexual violence perpetrated by partner.	Women who had experienced IPV had increased odds of having undergone a TOP (adjusted OR 2.68, 95% CI 2.34 to 30.6).	26
Antai and Adaji 2012 [73]	19,226 women aged 15–49 y. Demographic and Health Survey; Nigeria.	Physical, sexual, or emotional violence perpetrated by current or former partner.	Lifetime prevalence of IPV among women who had undergone a TOP: 21% physical violence; 6% sexual violence; 19% emotional violence.	26
Stöckl et al. 2012 [74]	3,270 women recruited from several districts within Tanzania. WHO Multi-Country Study on Women's Health and Domestic Violence against Women; Tanzania.	Lifetime physical and/or sexual IPV (perpetrated by a partner).	Women who report having experienced both physical and sexual IPV are more likely to have undergone a TOP than those who do not.	24
Alio et al. 2011 [75]	2,570 women aged 15–49 y. Demographic and Health Survey; Cameroon.	Physical, sexual, or emotional IPV in last year.	OR_adj_ for TOP: 1.59 (95% CI 1.10 to 2.31) with physical violence; 1.87 (95% CI 1.23 to 2.83) with sexual violence; 1.43 (95% CI 0.98 to 2.08) with emotional violence.	27
Okenwa et al. 2011 [76]	Nationally representative sample of women of reproductive age (*n* = 33,385). Demographic and Health Survey; Nigeria.	Exposure to physical, emotional, and/or sexual IPV over past 12 mo.	Women who had undergone TOP, miscarriage, or stillbirth were more likely to have experienced physical, sexual, and/or emotional violence than women who had not undergone TOP (*p*<0.001). Rates of violence among women who had undergone TOP, miscarriage, or stillbirth as compared to those who had not differed as follows: 20% versus 14%, 6% versus 3%, and 30% versus 22% for physical, sexual, and emotional abuse, respectively.	26
Emenike et al. 2008 [77]	5,878 women aged 15–49 y resident in or visiting households; Demographic and Health Survey; Kenya.	Lifetime history of physical, emotional, and/or sexual violence.	Women exposed to physical, emotional, or sexual violence were more likely to have experienced a TOP (*p*<0.001).	25
Kaye et al. 2006 [78]	Women presenting with abortion complications (miscarriage or TOP) (miscarriage *n* = 609; TOP *n* = 333); Uganda.	IPV during pregnancy.	IPV during pregnancy was a risk factor for TOP (OR 18.65, 95% CI 11.23 to 30.96, standard error 4.823, *p*<0.001).	24
Kaye et al. 2005 [79]	Women presenting with abortion complications (miscarriage or TOP) (miscarriage *n* = 609; TOP *n* = 333); Uganda.	Physical or sexual IPV during pregnancy.	Most common reason for TOP among adolescents and older women was “relationship issues” (including IPV). Domestic violence associated with TOP: point estimate 18.42, 95% CI 11.09 to 30.58, *p*<0.0001.	20
Kaye 2001 [80]	Every third women seen in a given time period with complications of TOP or miscarriage (*n* = 311); Uganda.	Physical, emotional, and sexual violence.	38.9% of women who reported TOP stated IPV as reason for choosing to terminate pregnancy.	14

OR_adj_, adjusted odds ratio.

**Table 7:** Characteristics of included cross-sectional studies from Asia.

Study	Population; Country	Exposure	Outcome	CASP Score (/30)
Nair et al. 2013 [81]	220 women living in slums who reported both IPV and a partner who had risky alcohol use; India.	30-d history of spousal physical or sexual violence.	11 of 77 [14%] women who reported IPV in the past 30 d had undergone a TOP, compared to 23/143 [16%] women not reporting violence in the past 30 d.	19
Nguyen et al. 2012 [82]	1,281 women in four districts of the Thai Nguyen province; Viet Nam.	Lifetime physical, sexual, and emotional gender-based violence based on the WHO definition.	Among women reporting any violence, 40.93% reported having undergone a TOP, compared to 30.54% of women not reporting violence (*p*<0.001). Results for physical, sexual, and emotional violence individually were also significant.	25
Shah et al. 2011 [83]	43 women who presented to a Pakistani hospital with complications of an unsafe TOP; Pakistan.	Physical and/or emotional violence. Time frame not specified.	Physical and/or emotional violence was given as a reason for TOP in 17.2% of cases.	13
Kalyanwala et al. 2010 [84]	549 unmarried, young women seeking TOP in Bihar and Jharkhand; India.	Forced or persuaded to have sex.	One in six participants stated that their pregnancy was the result of forced sex. Women forced to have sex were more likely to have a second trimester TOP.	19
Lee-Rife 2010 [85]	2,444 women aged 15–39 y living in India with at least one child. Selected by randomised household probability sample surveying; India.	Physical violence (hitting, slapping, kicking, beating, weapon use) perpetrated by husband from time of marriage to birth of first child.	Women who had had TOPs had higher odds of experiencing IPV (OR_adj_ 3.74).	16
Silverman et al. 2007 [86]	National sample of Bangladeshi women (*n* = 2,677) of childbearing age, married and living with their husband. Their husbands were also included; Bangladesh.	Husband asked about his perpetration of forced sex and physical IPV towards his current wife.	Women experiencing physical (but not sexual) IPV were at increased risk of having undergone a TOP in the past 5 y (OR_adj_ 1.54); women experiencing physical and sexual IPV were at increased risk of having undergone a TOP at any stage (OR_adj_ 1.43).	24
Leung et al. 2005 [87]	Patients requesting TOP (*n* = 300), infertility treatment (*n* = 500), obstetric care (*n* = 514), or general gynaecology treatment (*n* = 300) at a Hong Kong hospital; China.	Physical health, psychological health, social relationships, and environment assessed.	Obstetric and TOP patients showed significantly higher prevalence of lifetime violence compared to the other two groups (*p*<0.001). Most of these patients described emotional or verbal abuse. Quality of life was significantly reduced in all domains—physical, social, environmental, and psychological health (*p* = 0.014, 0.027, 0.002, and <0.001, respectively). It was noted that women reporting IPV were significantly more likely to be single or separated than married, with 35.0% of the 117 women reporting IPV being single or separated, compared to only 14.9% of 1,497 women not reporting IPV.	23
Wu et al. 2005 [88]	Women who were requesting a TOP and who had lived in the local city for at least 1 y (*n* = 1,215); China.	Physical, emotional, or sexual violence occurring during or prior to current pregnancy.	Lifetime experience of IPV was 22.6%. 2.1% of women stated that their current partner was forcing them to have a TOP. 14.6% of women reported that they were afraid of their partner. Women who had been abused were at significantly higher likelihood of multiple TOPs than women who had not been abused (*p*<0.001, OR 1.7, 95% CI 1.41 to 2.67). No significant difference in age between the two groups was noted, with average age in the group reporting IPV being 24.8±6.4 y, as compared to 25.7±6.4 y in the group not reporting IPV.	23

Percentages in brackets are calculated percentages not reported in the original studies.

OR_adj_, adjusted OR.

**Table 8:** Characteristics of included cross-sectional studies from Australasia.

Study	Population; Country	Exposure	Outcome	CASP Score (/30)
Fanslow et al. 2008 [89]	Random sample of 2,855 women aged 18–64 y, obtained from Auckland and Waikato; New Zealand.	Physical and/or sexual IPV perpetrated by husband, a man that the woman had lived with, or current, regular male sexual partner.	Controlling for other variables, women who had experienced IPV were 2.5 times more likely to report a TOP than those who had never experienced IPV (21.4% versus 9.9%, *p*<0.0001).	24
Whitehead and Fanslow 2005 [90]	125 women who agreed to see a social worker whilst attending an abortion clinic; New Zealand.	Lifetime and past year histories of physical abuse and sexual abuse (forced or pressured into having sex).	Reported lifetime prevalence of physical or sexual abuse was 50.8%. 69% of women who reported a lifetime history of physical abuse also reported that their partner/father of pregnancy was a perpetrator of their abuse. 42% reported that a family member was responsible.	18
Taft et al. 2004 [91]	Cohort of Australian women aged 18–23 y who are part of a long-term longitudinal study (*n* = 14,784). Survey 1 from prospective cohort study; Australia.	1-y history of physical and/or sexual violence. Lifetime history of violent relationship.	Lifetime partner violence was strongly associated with the following pregnancy outcomes: miscarriage and TOP (*p*<0.001) and birth, miscarriage, and TOP (*p* = 0.05). Recent partner violence was associated with the second of these outcomes (*p*<0.001). Neither situation was significantly related to TOP alone. Women reporting IPV were less likely to be married than single (OR 0.70, 95% CI 0.59 to 0.83), and more likely to be separated, divorced, or widowed than single (OR 2.62, 95% CI 1.78 to 3.86).	20
Webster et al. 1996 [92]	1,014 women seeking pregnancy care in Brisbane hospital; Australia.	Historic abuse (victim was >16 y old but abuse ended before current pregnancy began) and current pregnancy abuse. Physical, emotional, and sexual abuse studied.	29.7% of women disclosed past or present abuse history. Women reporting abuse were significantly more likely to have had a previous TOP than women who reported no history of violence (*p* = 0.0034).	22

**Table 9:** Characteristics of included qualitative studies.

Study	Population; Country	Exposure	Outcome	CASP Score (/20)
Kalyanwala et al. 2012 [93]	26 unmarried, young women seeking TOP; India.	Forced sex, or persuasion into having sex.	Those who reported that their pregnancy was the result of an incident of forced sex often reported fear of, as well as real, family violence.	14
Puri et al. 2011 [94]	65 women who had migrated from the Indian subcontinent to the US at the age of 18 y or greater, and had a history of seeking sex selection services; US.	Any marital violence related to fetal sex and/or sex selective TOP.	62% of women described verbal abuse from their female in-laws or husband; one-third described past physical abuse and neglect related specifically to their failing to produce a male child.	14
Williams and Brackley 2009 [95]	8 women aged 18–45 y with a self-reported history of IPV within the past year, or since becoming pregnant, presenting for TOP (unintended pregnancy) and who could read, write, and comprehend English; US.	Self-reported IPV within past year, or since becoming pregnant.	Researchers identified consistent themes within abuse patterns: women reported that the violence was “not that bad” initially, it then escalated, and, finally, they believed that if they were to carry their pregnancy to term, their partner would return.	12
Belton 2007 [96]	Burmese women migrating to Thailand, living in the Tak province. 180 case notes reviewed; 31 public hospital case notes reviewed; 43 women and 10 men interviewed; case notes of 14 women who died during or shortly following obstetric care reviewed; 20 midwives interviewed; Thailand.	Relationship with father of pregnancy; fear of father of pregnancy.	5/43 [12%] women reported IPV as a motivation to end their pregnancy. 3/10 [30%] men disclosed controlling, threatening, or physically abusive behaviours against their wife.	10
Renker 2002 [97]	139 women aged between 18 and 19 y who were pregnant; US.	Physical violence in the lead-up to and during a pregnancy.	40/139 [29%] pregnant teenagers identified as having been abused in the years leading up to their current pregnancy, and 13 of these 40 [33%] reported pregnancy in the same year that ended in miscarriage or TOP.	10
Souza and Ferreira 2000 [98]	12 women attending a local hospital for post-TOP care; Brazil.	Physical, sexual, and emotional violence.	Although all women accepted the definitions of IPV they were shown, and some identified that they had experienced such activities, none of the participants answered positively when asked directly whether or not their partner had acted violently towards them.	9

Percentages in brackets are calculated percentages not reported in the original studies.

### Key Features of Studies

The publication dates of the studies ranged from 1985 to 2013, with the majority (67/74, 91%) having been reported since 2000. Sixty-eight studies included quantitative data, with six being exclusively qualitative. The majority of quantitative studies were cross-sectional (57), with the remaining being cohort (three) and case-control (eight, of which five used a pregnant and three used a non-pregnant comparator group). Geographically, the study locations spanned six continents: North America (35), Asia (12), Europe (10), Africa (8), Australasia (6), and South America (2), with one further study analysing data from several continents. Women were asked about IPV in a variety of settings, including home, gynaecology wards, termination clinics, and other specialist medical clinics, and in various ways, including telephone or written questionnaire and national scale surveys. Among the quantitative studies there were 10 high, 31 medium, 19 low, and eight very low quality reports (mean score 20.2, standard deviation 4.0). Of the qualitative studies two were medium, one low, and three very low quality (mean score 11.5, standard deviation 2.2). The majority of studies analysed exclusively women (69), with few analysing exclusively men (two) or both men and women (three). Sample sizes ranged from eight to 33,385 participants (eight, 30, and 36 studies had <100, 100–999, and ≥1,000 participants, respectively; median number of participants 942, interquartile range 208 to 2,391). The exposures included physical violence (53), sexual violence (47), and emotional violence (19), with many articles looking at combinations (42). Table S5 shows the associations of clinical and demographic factors with IPV in women seeking TOP.

### Prevalence and Meta-Analysis of Lifetime Prevalence

**Figure 2:**
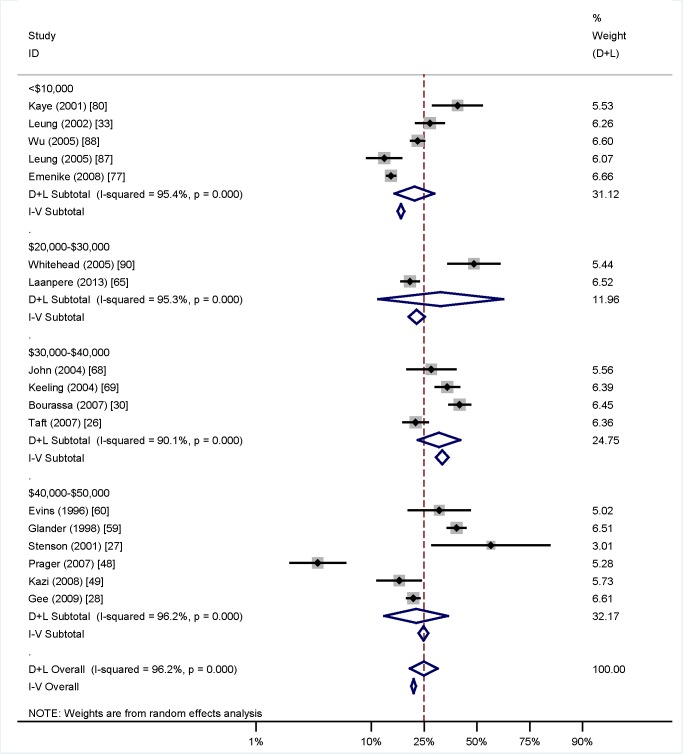
Prevalence of intimate partner violence among women seeking termination of pregnancy grouped by country's gross national income per capita (in intervals of Int$10,000). Weights are from random effects analysis. D+L, combined effects using the DerSimonian and Laird [21] random effects method; I-V, combined effects using the inverse variance fixed effects method.

**Figure 3:**
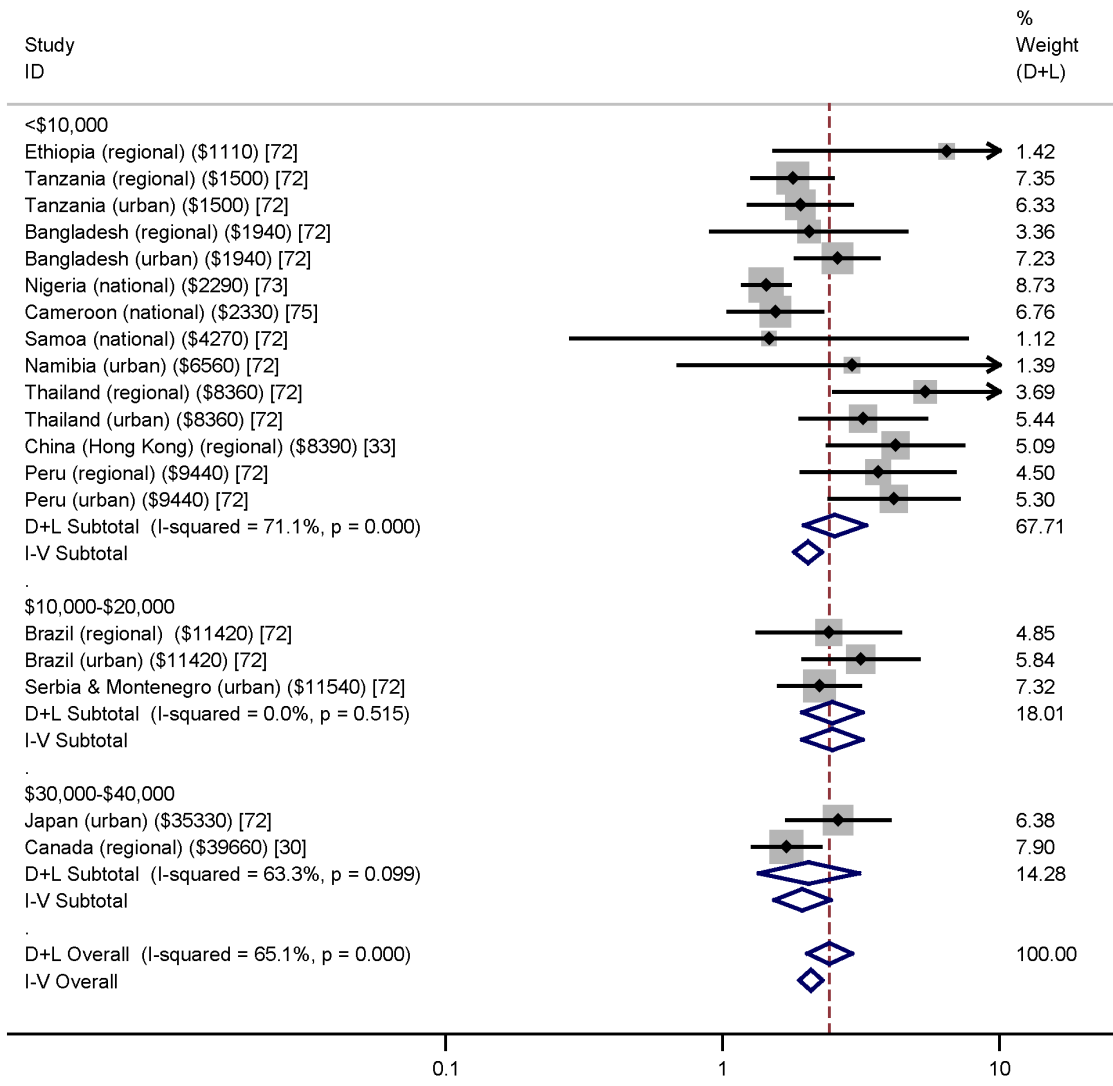
Associations between intimate partner violence and termination of pregnancy grouped by country's gross national income per capita (in intervals of Int$10,000) and with setting (urban, regional, or national) given. Weights are from random effects analysis. D+L, combined effects using the DerSimonian and Laird [21] random effects method; I-V, combined effects using the inverse variance fixed effects method. Countries grouped by GNI (shown in parentheses).

Among women who underwent TOP, reported rates of IPV in the preceding year ranged from 2.5% [43] to 30% [76], while lifetime rates of IPV in this population varied from 14% [88] to 40% [59]. Meta-analysis of lifetime prevalence of IPV among TOP-seeking populations was found to be 24.9% (95% CI 19.9% to 30.6%), as shown in Figure 2. Following systematic meta-analysis regression, we found that the high level of variation between studies was not explicable by study quality, type, or size, or country GNI per capita (Table S6). The association between IPV and TOP is shown in Figure 3; variation is not explained by GNI per capita. *I* ^2^ values for heterogeneity were high, as expected when pooling data with proportions.

### Meta-Analysis of Risk Factors

**Figure 4:**
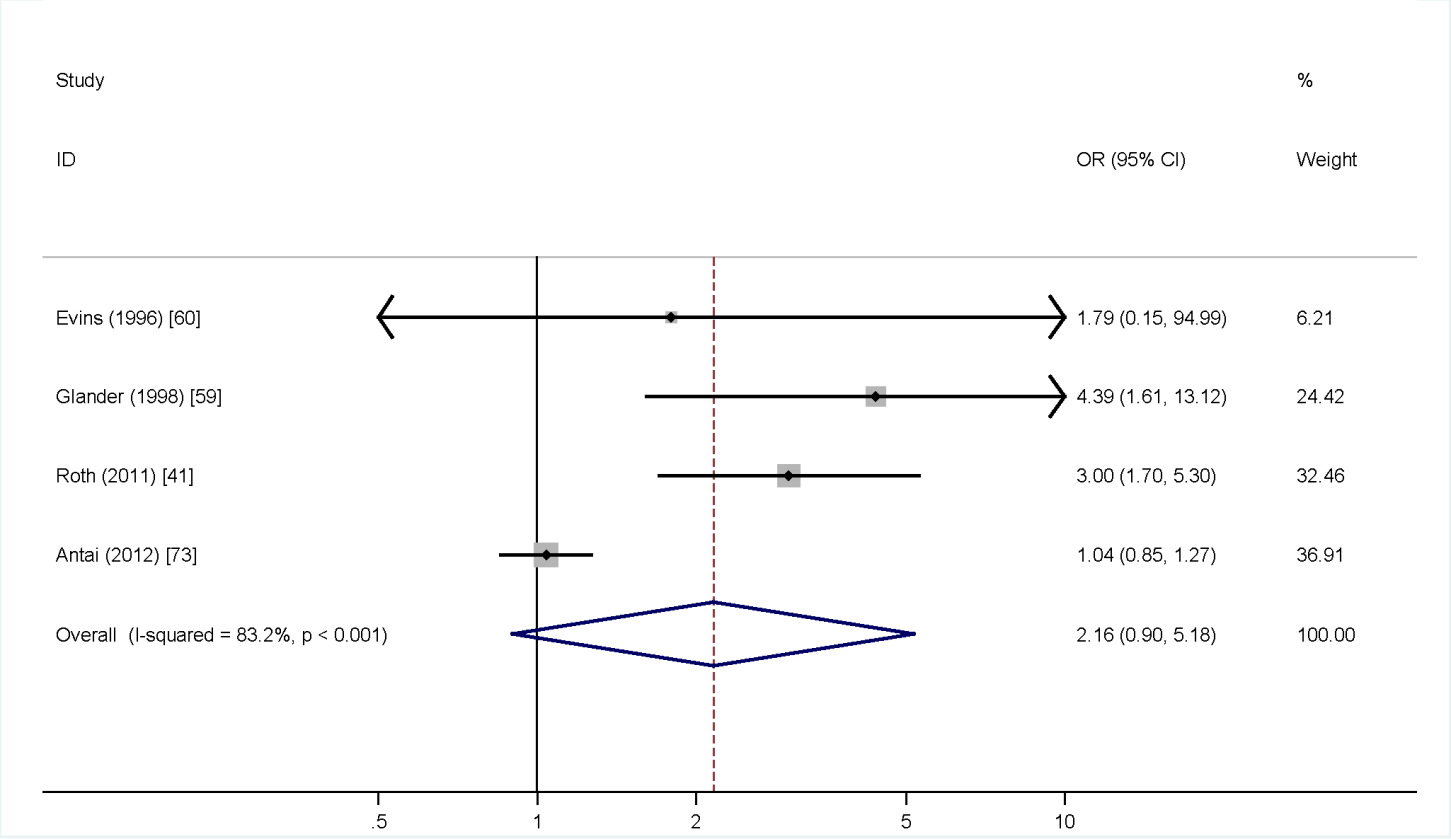
Single status and intimate partner violence. Weights are from random effects analysis. OR, odds ratio.

**Figure 5:**
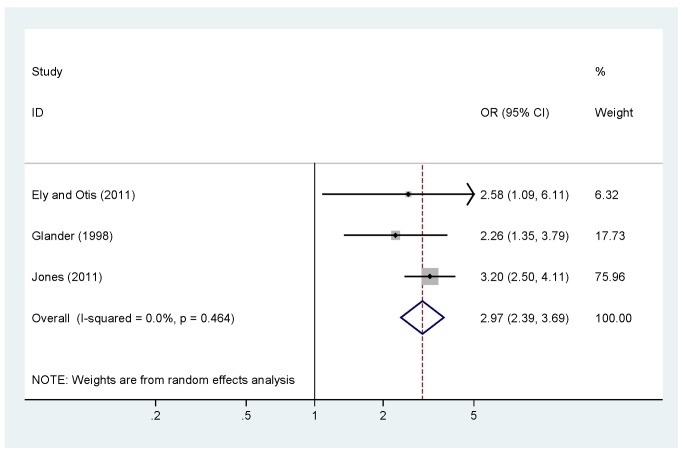
Partner knowledge of termination of pregnancy and intimate partner violence. Weights are from random effects analysis. OR, odds ratio.

**Figure 6:**
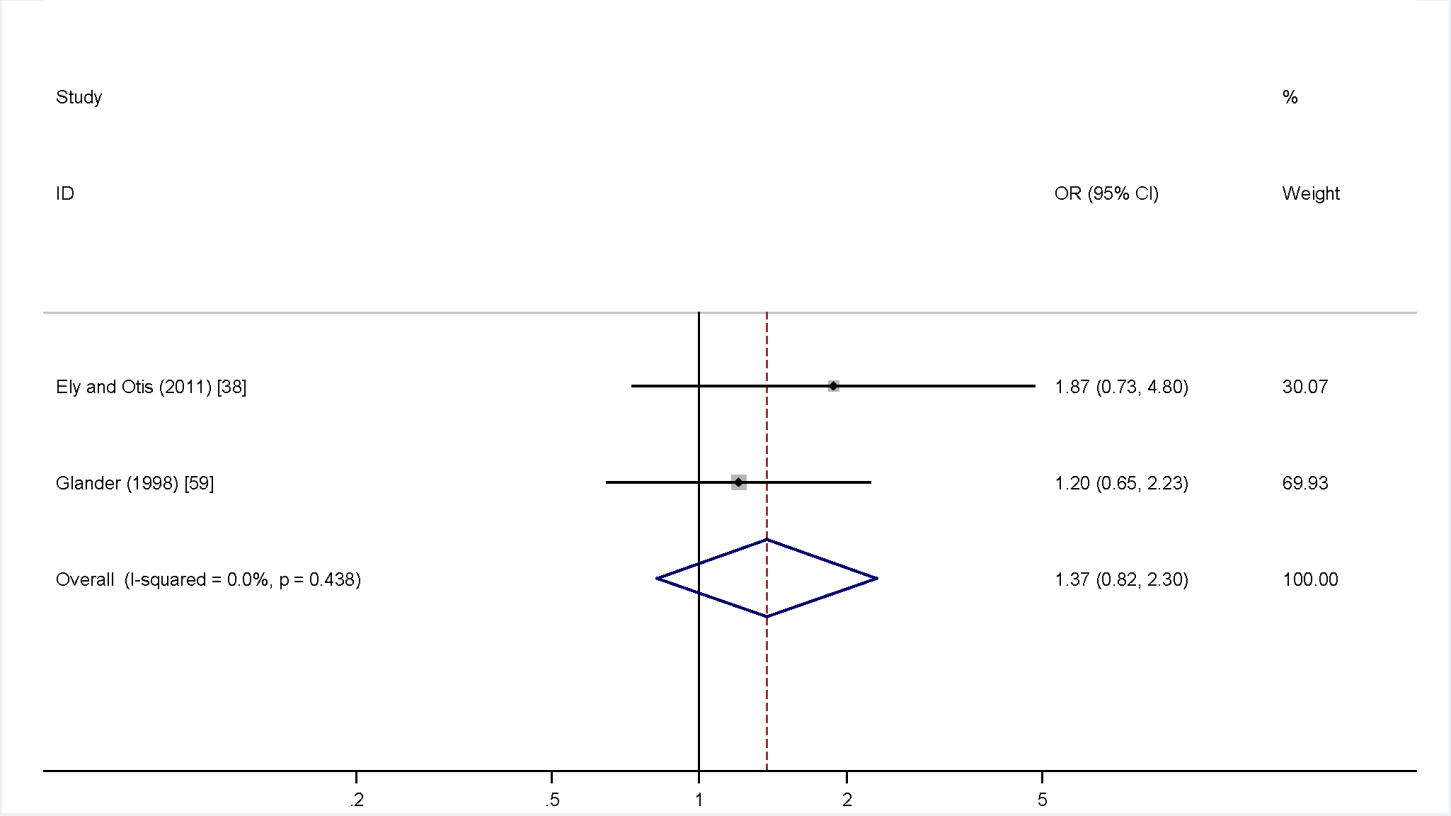
Partner support for termination of pregnancy and intimate partner violence. Weights are from random effects analysis. OR, odds ratio.

**Figure 7:**
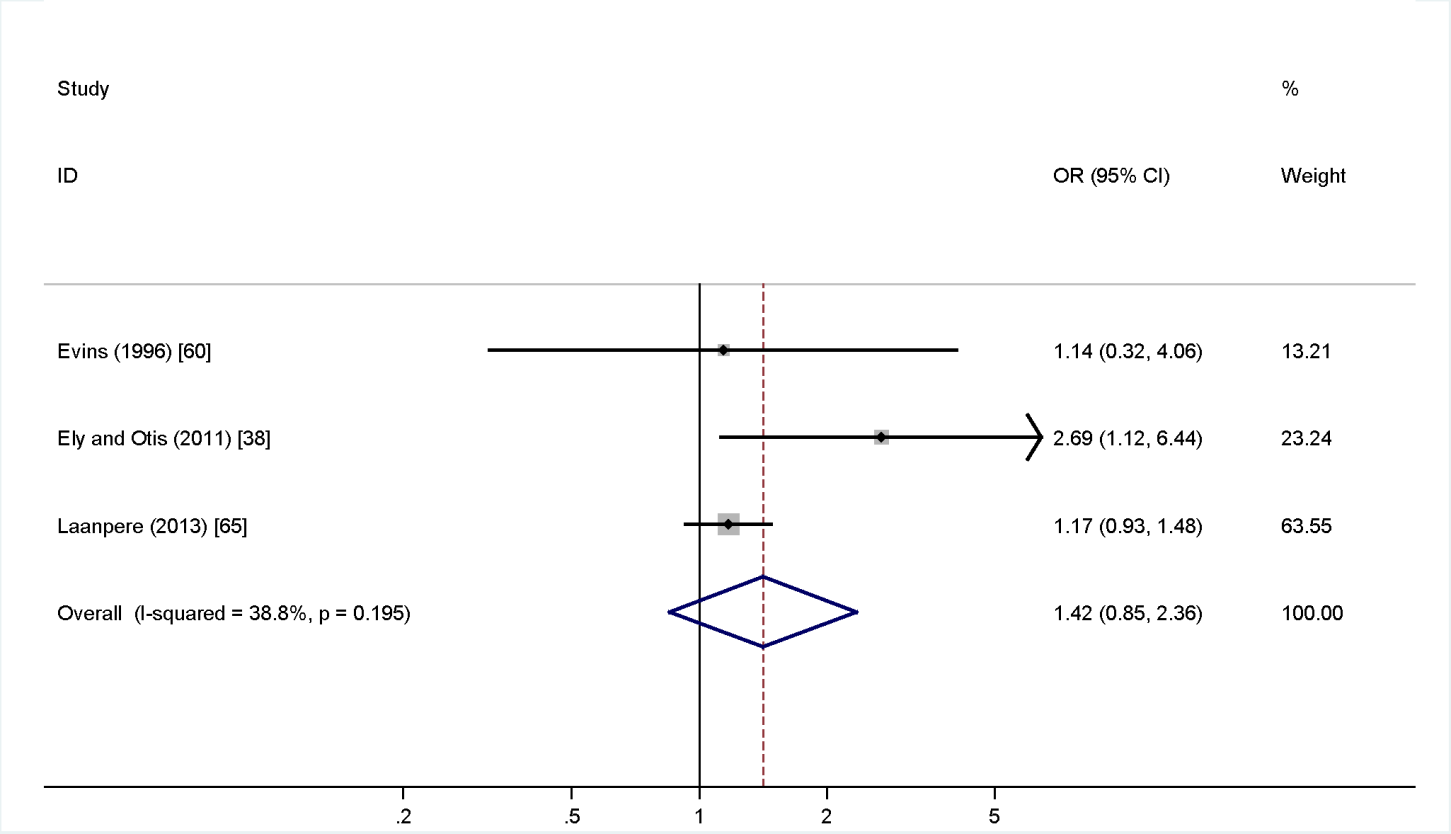
Previous terminations of pregnancy and intimate partner violence. Weights are from random effects analysis. OR, odds ratio.

Meta-analysis was undertaken for four factors where data were available (woman being single versus married, partner not knowing about the TOP, partner support for the TOP, and previous TOP), but was not possible for others, as the definitions of both IPV and the risk factors varied, or there was a lack of numerical data or only a single study in many cases. Figure 4 shows no association (pooled OR 2.97, 95% CI 2.39 to 3.69) between being single and IPV among a TOP-seeking population. There was an association (pooled OR 2.32, 95% CI 2.00 to 2.69) between partner not knowing about the TOP and IPV (Figure 5), but no association between partner support for the TOP and IPV (pooled OR 1.37, 95% CI 0.82 to 2.30) (Figure 6). Meta-analysis of three studies reporting on the association between IPV and previous TOP showed no significant association (pooled OR 1.42, 95% CI 0.85 to 2.36) (Figure 7), but five of six further studies reported individual significant associations, though with inadequate quantitative data to include in the meta-analysis. Table S5 shows the associations of clinical and demographic factors with IPV in women seeking TOP.

### Associations with Reproductive and Pregnancy Factors

#### Past obstetric history

Nine studies showed that women who reported IPV were more likely than the comparator group to have a history of multiple TOPs (Tables 3–5) [27],[28],[38],[41],[42],[44],[51],[60],[65]. The highest quality study found that women presenting for a third TOP were over two and half times more likely to have a history of physical or sexual violence than women presenting for their first [51]. There was no significant association between number of pregnancies and IPV (gravidity in group of women reporting IPV: mean 3.2, standard deviation 2.0; among women not reporting IPV: mean 3.0, standard deviation 2.0) [41], although one medium quality study found a higher odds of history of previous miscarriage among women reporting IPV at a termination clinic, as compared to those not reporting IPV (OR 1.6, 95% CI 1.1–2.2) [41].

#### Coercion, contraception, and conception

One study investigating pregnancy intention found that women in violent relationships were more likely to say that the pregnancy “had been imposed” upon them by their partner (13% versus 2% for women not in physically or sexually violent relationship) [29]. A further two studies found that pregnancy associated with sexual coercion and ending in TOP was also 1.8- to 3.8-fold more likely to be associated with IPV [34],[35]. One study found that women in violent relationships were significantly more likely to report going without contraceptives compared to women not reporting IPV (Table 3) [28], with another study finding that they were more likely to use them (Table 4) [38]. In four studies, of which two were very low quality, women reported being actively prevented from obtaining contraception by their partner, or that their partner was refusing or deceiving them about birth control use (Tables 3 and 4) [28],[38],[45],[52]. It was reported that a partner preventing access to contraception led to concealed use of contraceptives among some women [45].

One cross-sectional population telephone survey of over 4,000 women found that over 46% of 616 “completed rapes” were perpetrated by a husband or boyfriend, and that 50% of 20 rape-related pregnancies ended in TOP [61]. One low quality study of women who migrated as adults from the Indian subcontinent to the US and had a history of seeking sex selection services found that a third of 65 women described past physical abuse and neglect related specifically to not producing a male child [94].

### Factors Relating to Termination of Pregnancy

#### Decision-making

Women reporting IPV were more likely to report an unwanted pregnancy (7.4% of 163 women not reporting IPV versus 23.3% of 44 women reporting IPV) [53]. Additionally, a high quality study, though with small numbers, found that women attending a termination clinic with a planned pregnancy were more likely to report IPV (50% of 12 women) than those who did not plan their pregnancy (5.6% of 337 women) [30]. There was evidence from very low quality studies that some women felt coerced by their violent partner into having a TOP (Table 4) [45],[52]. One medium quality study found that 2% of 1,215 women in a termination clinic reported being forced into the decision by their partner [88]. One small, very low quality study of 38 women participating in a hospital-based IPV programme found that 18% reported feeling “pressured” into TOP and 5% were forced into undergoing the procedure [52].

There was consistent evidence that women in violent relationships were more likely not to tell their partner about their decision to terminate (pooled OR 2.97, 95% CI 2.39 to 3.69; Figure 5) [38],[39],[54]. Women with an IPV history were less likely to have their partner fund their TOP (27% of 25 women reporting IPV had their partner pay for their TOP versus 63% of 155 women not reporting IPV) [38].

#### Gestation and method of termination of pregnancy

Two studies, one of high and one of low quality, failed to find any association between IPV and gestation at TOP [54],[57], although one lower quality study reported that women having a second trimester TOP were more likely to report a history of forced sex than women having a first trimester TOP (35.3% of 410 versus 11.5% of 139), [84], and another found that women later in their second trimester (over 16 versus 13–15 weeks' gestation) at time of TOP were more likely to report IPV (OR 1.23, 95% CI 0.79 to 1.91) [40]. One further study found that women who reported IPV were more likely to have the gestational age of the fetus redated on ultrasound (64.9% of 243 women reporting IPV versus 49.9% of 817 women not reporting IPV) [41].

#### Psychosocial problems

In women who had undergone TOP, there was a significant association between reported IPV and psychosocial problems including depression [38],[47], suicidal ideation [47], stress [38],[47], and disturbing thoughts [47], although the temporal relationships were unclear (Table 4) [38],[47]. No studies identified the subsequent impact of IPV and TOP on the woman, her partner, or their relationship.

#### Disclosure and intervention

Five studies examined disclosure in termination clinics: IPV questionnaires were highly acceptable [90], although non-responding women differed from those who responded and had undergone more TOPs [41]. Women in violent relationships were as likely to attend for follow-up (52.2% of 413 non-abused women defaulted versus 46.6% of 88 abused women) [33] and more likely to know about community resources for IPV (80% of 16 women reporting IPV versus 67% of 35 women not reporting IPV) [60]. Some women reported events that would meet the definition of IPV but did not identify themselves as experiencing IPV [98]. Nevertheless, many women wished to talk about IPV with regard to further management or intervention [33], with some citing their doctor as the main source of information [60]. However, during a period of universal screening only 51% of 499 women were asked about IPV; certain sub-groups of women were more likely to be asked about IPV (e.g., white women, in a Canadian study, in which white women made up 55% of the TOP-seeking population [*n* = 499], but 63% of those asked about IPV [*n* = 254] [57]).

### Demographic Factors

#### Female factors

The majority of studies focused on female factors. The results relating to impact of age on the association between IPV and TOP were discrepant, and interpretation is hindered by different age groups being used. One paper reported that past year incidence of IPV was higher among women under the age of 20 y seeking TOP (50.0%) than among those 20 y or over (26.9%); however, the converse [29],[30] or no significant association (Tables 4, 6, and 7) [41],[57],[75],[88] has also been reported.

There was contradictory evidence for variance between women of different race or ethnicity, with two studies finding converse associations; one found that significantly fewer white Caucasian women (12% of 160 women) reported IPV as compared to non-white women (21% of 94 women, *p* = 0.003) [57]; another study of women seeking elective pregnancy termination reported that a greater proportion of white women (48% of 226) had experienced IPV compared to black women (31% of 223, *p* = 0.001) [59]. This finding may not be generalisable to other locations. No significant associations were found with women's level of education in two studies [41],[88] or with women's income [41]. Three studies reported no significant association with employment status [41],[69],[88], whilst two suggested that unemployed women or non-skilled labourers were more likely to report both IPV and TOP (25.3% of 291 women seeking TOP and reporting IPV were unemployed versus 19.1% of 1,096 women seeking TOP and not reporting IPV) [28],[33].

Three studies of similar medium quality assessed drug and alcohol use; one study found that, compared to women not reporting IPV, women in a termination clinic who reported IPV were also more likely to smoke (25.6% of 117 women experiencing IPV versus 11.0% of 1,497 non-abused women) and drink (12.8% of women experiencing IPV versus 4.9% of non-abused women) [87], but other studies found no significant association [88] or reported an association only anecdotally [59].

An association between negative physical quality of life scores and IPV and TOP was found in one medium quality study (Table 7) [87].

#### Relationship factors

Five studies, one of high and four of medium quality, found that, compared to married women, those who were single, separated, divorced, or widowed were more likely to have a history of IPV and TOP (Tables 3, 4, and 7) [30],[41],[43],[87],[91], though one medium quality study found no significant association [88], and one further study of women experiencing IPV found married women were more likely to report a TOP (89% of 23,909 married women compared to 11% of 9,408 unmarried women) [73]. Women who reported difficulties in their relationship were also more likely to report IPV when asked at a termination clinic in all studies (with one study finding that 7.7% of 350 women undergoing a TOP reported difficulties in their relationship, as opposed to 1.8% of 653 women continuing pregnancy) [30],[38],[59]. One study found no significant association between prevalence of IPV and household income (27.2% of 669 women reporting TOP were in the lowest income bracket, as compared to 26.5% of 139 women not reporting TOP) [54].

#### Male factors

One article studied men attending community health centres and identified that men who admitted to IPV perpetration were more likely to report having “been involved in a pregnancy” that ended in TOP (48.9% of 188 men reporting IPV perpetration also reported involvement in TOP, as opposed to 22.7% of 402 men not reporting IPV perpetration) [44]. Another study of partners of women having a first or subsequent TOP found higher rates of IPV experienced by the men involved in a second or more TOP (12% of 188 men whose partner was having a second or subsequent TOP reported “being a victim” of IPV versus 6% of men whose partner was having a first TOP) [66]. One low quality study noted that 36% of 16 women reporting IPV stated that their perpetrating partner had been the victim of abuse as a child [60].

**Figure 8:**
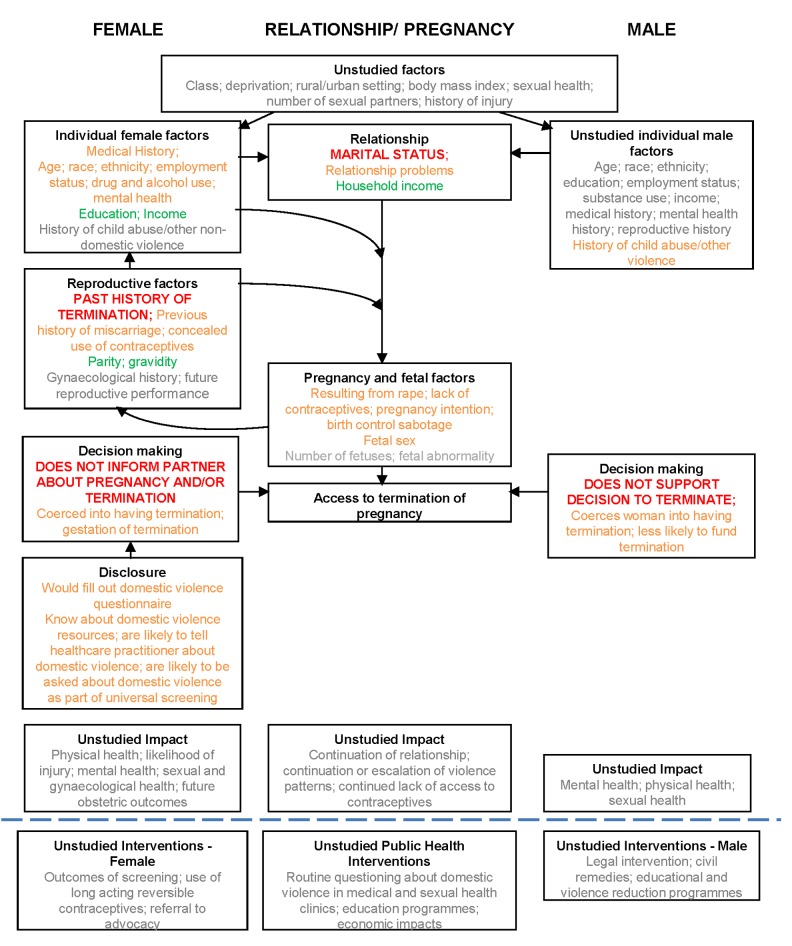
Matrix of associations between domestic violence and termination of pregnancy. Key to associations: red, associations meta-analysed; amber, associations not meta-analysed but shown in literature; green, no significant association described in the literature; grey, not studied.

For IPV in women presenting for TOP, there was no evidence of bias relating to non-publication of small non-significant studies; Egger's test [24] for small-study effects was non-significant (*p* = 0.13). These associations, the uncertainties, and gaps in knowledge are shown diagrammatically in Figure 8.

There were no intervention studies.

## Discussion

### Summary of Main Findings

The literature is extensive, but variable in quality, and largely focused on female factors. High rates of physical, sexual, and emotional IPV were found across six continents among women seeking a TOP. According to meta-analysis, partner not knowing about the TOP was associated with IPV among women seeking TOP. However, lack of partner support for TOP is not associated with IPV among women seeking TOP. The literature also suggests that women in abusive relationships were more likely to report inability to make autonomous contraceptive choices, partner contraceptive sabotage, and sexual violence, and they were less likely to have informed their partner about the pregnancy or involved him in decision-making about it. IPV was cited as a reason for wanting TOP, and rape-related pregnancy had a particularly high chance of leading to TOP. The meta-analysis did not find that multiple TOPs were associated with IPV, but its credibility is undermined by both the number of studies that did (albeit unquantified) and the association reported by men who stated they had been perpetrators of IPV. Although it was not always determined whether experiencing IPV was a determining factor in the decision to end, rather than continue, a pregnancy, the findings support the concept that violence can sometimes lead to an initial pregnancy (via coercion, rape, sexual assault, or contraceptive sabotage) and to a subsequent TOP (via coercion). There was a lack of data regarding long-term outcomes for women in violent relationships who underwent TOP, but associations with repeat TOP (and possibly miscarriage) lend support to the notion of a repetitive cycle of abuse and pregnancy. Not informing the male partner may then be explicable as a reason to avoid partner involvement or further abuse.

### Strengths and Limitations

Strengths of the review and meta-analysis include use of multiple databases, no language restriction, hand searching of reference lists, double data entry, and quality assessment of both quantitative and qualitative studies from a wide variety of settings, thus improving reliability and generalisability. Limitations include unexplained large differences in both prevalences and odds ratios between studies (heterogeneity), likelihood of underreporting of both IPV [99] and TOP (particularly when both are stigmatised) in the primary data sources, and inherent difficulties in validation. There is potential publication bias towards research showing a positive relationship. The meta-analysis may be biased, as only those studies quantitatively reporting a significant result could be included, whilst those stating an association (without providing data for inclusion) had to be excluded. It was not possible to determine the legality of, or access to, TOP for each country or state at the specific time point of study; thus, the analysis has not included evaluation of such barriers. It was not possible to determine temporal relationships and patterns of abuse, pregnancy, and TOP.

### Comparison with Other Studies

Only one review has previously examined the association between IPV and TOP within a study of broader sexual health issues [100], concluding that TOP and repeat TOP were associated with IPV, but without reporting on other associations. The review was single-authored, lacked systematic analysis, and included only eight studies. Other relevant systematic reviews and observational studies on IPV have included women with ongoing pregnancies, women with pregnancy loss, or all women of reproductive age, noting poorer mental, physical, and pregnancy outcomes for the women experiencing IPV [101]–[104]. One review found an association between presence of depressive or anxiety disorders in women and increased likelihood of IPV compared to women without mental disorders, but the direction of causality could not be determined, as few studies were longitudinal [104]. IPV was also associated with physical injury: presentation to hospital accident and emergency departments with unwitnessed head, neck, or facial injuries was a significant marker for IPV [103]. A large observational study conducted by WHO found that women who reported a history of partner violence were more likely to report physical and mental health problems including emotional distress and suicidal thoughts, as well as difficulties with activities of daily living [101]. Comparison groups that might prove useful would be women with ongoing but unplanned pregnancy (no specific studies available) and women with ongoing wanted pregnancy. In ongoing pregnancies, IPV was associated with 1.5-fold increased risks of low birth weight and preterm birth [102]. A large observational study has reported poorer maternal outcomes, including hypertension, renal and urinary tract infection, and vaginal bleeding, with IPV [8].

### Implications and Clinical Relevance

Health-care professionals should be aware of the high rates of physical, sexual, and emotional violence among women seeking TOP, and particularly the clinical factors associated with greatest risk: previous TOP, lack of contraception, initially planned pregnancy, ultrasound redating, and the partner not funding or not being told about the TOP. There are potential associations for IPV with young age, marital status, ethnicity, and low household income. IPV compromises both the safety and health of the woman requesting the TOP, and potentially that of her partner and any existing children if a woman retaliates or children witness or experience the violence directly. In attempting to prevent repeat TOP [105], a narrow focus, especially on long-acting contraception, that excludes addressing the wider needs of a woman in a violent relationship might leave a woman less likely to become pregnant but just as vulnerable to IPV. Good practice obligates that termination services should have robust policies for ensuring women's safety and confidentiality, providing information and referral pathways for those who disclose IPV, and exemplar guidance exists [16].

Some groups have evaluated whether screening for IPV is justified in selected populations of women. Theoretically, early identification and effective intervention for violence may reduce repeat unintended pregnancy and TOP, as well as improve longer term health outcomes. Three systematic reviews [106]–[108] have concluded that screening is warranted, leading the US Preventative Task Force to recommend that clinicians should screen women of childbearing age for interpersonal violence, such as IPV, and refer women who screen positive to intervention services [109]. The UK National Institute for Health and Care Excellence is currently undertaking a consultation regarding guidance on identification and prevention of IPV [110]. However, interventions in this area are inherently complex and difficult to research [111], and evidence for the effectiveness of counselling intervention programmes or other interventions remains limited [106],[112],[113]. Of all approaches evaluated, intensive advocacy (aiming to provide women with information and support to facilitate access to community resources) appears the most promising in reducing physical abuse 1–2 y after the intervention, but impact on quality of life or mental health is not proven [112]. The majority of interventions studied have focused on the female experiencing IPV, but some have undertaken theoretical analysis of models in which changes in the behaviours of the male perpetrators are included [114]. A comparative study of promising health-based IPV interventions in primary care and maternity services across Europe found that key implementation issues of IPV interventions included clinical champions, leadership roles, funded and coordinated multiagency partnerships with clear referral pathways, multidisciplinary and participant feedback, and evaluation of outcomes [115]. Interventions as part of the pathway for women seeking TOP require further consideration.

### Future Research

No study we found set out to examine the association or temporal relationships between IPV and TOP, which would require (at a minimum) including women with and without an IPV history and with and without a history of TOP. There is extremely limited information about male partners of women seeking TOP, as perpetrators or as experiencing IPV, and which male-related factors contribute to increased likelihood of IPV. Greater information is required on long-term outcomes of violence and TOP on both partners. The findings of pregnancy “concealment” and higher rates of murder and suicide with IPV [116],[117] mean that researchers must be cautious and aware of women's safety. Harms have been identified following health-based IPV interventions, such as breaches of confidentiality [115]. Therefore, a public health approach that does not focus solely on the woman (either as “problem” or “solution”) or health services should be considered, for example, using educational, social norm, and/or criminal justice interventions. Nevertheless, given the clear associations, termination services provide an appropriate setting in which to assess screening for, or give information about, IPV, whether pre- or post-TOP, and for offering an intervention that women desire, such as a “one stop” offer of referral to specialist IPV services, especially in view of low return to clinics for follow-up [118]. Given that routine identification of women experiencing IPV and provision of a standard intervention has recently been shown to have no impact on quality of life or mental well-being, there is now a need for considering new strategies, including alternative intervention models and targeting perpetrators as well as the women affected [119]–[121]. On the basis of this review, research into the suitability, acceptability, and design of an intervention programme is justified, and should be tested preferably in a randomised control trial. Any legal barriers to intervention and reporting, such as criminalisation of TOP, should also be investigated and described.

### Conclusion

IPV is associated with TOP. Novel public health approaches are required to address IPV against women and repeat TOP. Termination services provide an opportune health-based setting in which to design and test interventions at the individual level.

## Supporting Information

Table S1 **Data extraction form.**
(DOCX)

Table S2 **Quantitative CASP form.**
(DOCX)

Table S3 **Qualitative CASP form.**
(DOCX)

Table S4 **PRISMA table.**
(DOCX)

Table S5 **IPV and associated factors.**
(DOCX)

Table S6 **Lifetime prevalence of intimate partner violence in women presenting for termination of pregnancy: meta-analysis regression to compare odds ratios between study categories.**
(DOCX)

Text S1 **Protocol.**
(DOCX)

## Abbreviations

CASP: Critical Appraisal Skills Programme
GNI: gross national income
IPV: intimate partner violence
OR: odds ratio
TOP: termination of pregnancy
WHO: World Health Organization
