# Supplementary material for: Associations between Intimate Partner Violence and Termination of Pregnancy: A Systematic Review and Meta-Analysis
Source: PLoS Med. 2014 Jan 7;11(1):e1001581. doi: 10.1371/journal.pmed.1001581 (PMC3883805; doi:10.1371/journal.pmed.1001581)
Supplement: Table S1 — Data extraction form. (DOCX) [file pmed.1001581.s001.docx]

**Table S1: Data Extraction Form**

| **Study Design** | RCT |  |
| --- | --- | --- |
|  | Prospective |  |
|  | Cross-sectional |  |
|  | Cohort |  |
|  | Case-control |  |
|  | Case series |  |
|  | Other (specify) |  |
| **Population** | Females seeking TOP |  |
|  | Females (specify) |  |
|  | Males (specify) |  |
| **Setting** | Termination clinic |  |
|  | Gynaecology clinic |  |
|  | Household clinic |  |
|  | Other (specify) |  |
| **Country/region** |  |  |
| **Intervention** |  |  |
| **Comparator population** |  |  |
| **Definition of domestic violence** |  |  |
| **Screening tool(s) used** |  |  |
| **Incidence/prevalence of domestic violence among** **women with termination** | Lifetime | N=.../... or ...% |
|  | Last year | N=.../... or ...% |
|  | Current pregnancy | N=.../... or ...% |
|  | Other time period | N=.../... or ...% |
| **Incidence/prevalence of termination among women with domestic violence?** | Lifetime | N=.../... or ...% |
|  | Last year | N=.../... or ...% |
|  | Current pregnancy | N=.../... or ...% |
|  | Other time period | N=.../... or ...% |
| **Were associations** **with any of the following sought? If YES, please describe:** |  |  |
| **Demography** | Age |  |
|  | Race/ethnicity |  |
|  | Marital status |  |
|  | Education |  |
|  | Class |  |
|  | Woman’s income |  |
|  | Household income |  |
|  | Depravation |  |
|  | Body mass index |  |
|  | Rural/urban |  |
|  | Perpetration of domestic violence |  |
|  | Substance use |  |
|  | Partner substance use |  |
|  | Relationship problems |  |
|  | Living arrangements |  |
|  | Employment status |  |
| **Reproductive health** | Parity |  |
|  | Gravidity |  |
|  | Gestation of TOP |  |
|  | Pregnancy intention |  |
|  | Previous obstetric history |  |
|  | Number of previous TOP |  |
|  | Partner knowledge of TOP |  |
|  | Financing of TOP |  |
|  | Coercion into having TOP |  |
|  | Sexual assault/rape |  |
|  | Future reproductive performance |  |
|  | Sexual health |  |
|  | Number of sexual partners |  |
|  | Use of contraceptives |  |
|  | Birth control sabotage |  |
| **General health** | Gynaecology history |  |
|  | Medical History |  |
|  | History of injury |  |
|  | History of child abuse/other non-domestic violence |  |
|  | Mental health |  |
| **Other** |  |  |
| **Was the impact of termination with or without domestic violence sought?** |  |  |
| **Was the impact of domestic violence with or without termination sought?** |  |  |
| **Were any domestic violence interventions assessed?** | Routine questioning |  |
|  | Referral information |  |
|  | Other |  |
