# Supplementary material for: Associations between Intimate Partner Violence and Termination of Pregnancy: A Systematic Review and Meta-Analysis
Source: PLoS Med. 2014 Jan 7;11(1):e1001581. doi: 10.1371/journal.pmed.1001581 (PMC3883805; doi:10.1371/journal.pmed.1001581)
Supplement: Table S2 — Quantitative CASP form. (DOCX) [file pmed.1001581.s002.docx]

**Table S2: Quantitative CASP Form**

**Author/Title/Journal**Scoring
0 – Study does not meet criteria/answer question
1 – Study partially meets criteria/gives a partially satisfactory answer to the question
2 – Study fully meets criteria/gives a fully satisfactory answer to the question

| Question | Considerations/Scoring Methods | Comments | Score |
| --- | --- | --- | --- |
| 1 – Does the study address a clearly focused question? | -focused in terms of population of interest  -focused in terms of outcomes studied |  |  |
| 2 – Is the study design appropriate to address the research question? |  |  |  |
| 3 – Does the study use an appropriate sampling method? | -sampling method  -time frame  -sample size |  |  |
| 4 – Is the study sample appropriate to address the research question? | -sample characteristics clearly described  -clear inclusion and exclusion criteria  - appropriate controls  -representativeness of sample |  |  |
| 5 – Is the level of non-participation tolerable? | -level of non-participation  - comparison of non-participants and participants  - impact of non-participation |  |  |
| – Is the exposure TOP/violence appropriately assessed? | -definition of TOP and violence is provided  -suitability of the indicators used  -potential for bias |  |  |
| 7 – Are the outcomes (violence or TOP) appropriately assessed? | -validated clinical and/or survey instruments used to assess outcomes |  |  |
| 8 – Are known confounders accounted for? | Key confounders not identified 🡪 0  Key confounders identified 🡪 1  Key confounders identified and included in multivariate analysis 🡪2 |  |  |
| 9 – Are appropriate statistical analyses conducted? |  |  |  |
| 10– Are prevalence/risk/odds ratio measures reported with confidence intervals? | Not reported 🡪 0  Reported without CIs 🡪 1  Reported with CIs 🡪 2 |  |  |
| 11– How precise are the results? | <100 participants 🡪 0  100-999 participants 🡪 1  ≥1000 participants 🡪 2 |  |  |
| 12 - Were ethical issues appropriately considered? | -informed consent  -safeguarding anonymity, confidentiality and safety  -availability of support and referral options  -fieldworker training |  |  |
| 13 - Do the findings support the conclusions? |  |  |  |
| 14 - Are the findings generalisable? | Not generalisable 🡪 0  Generalisable locally 🡪 1  Generalisable nationally 🡪2 |  |  |
| 15 - Study results fit with existing evidence |  |  |  |

Score: /30
