# Supplementary material for: Associations between Intimate Partner Violence and Termination of Pregnancy: A Systematic Review and Meta-Analysis
Source: PLoS Med. 2014 Jan 7;11(1):e1001581. doi: 10.1371/journal.pmed.1001581 (PMC3883805; doi:10.1371/journal.pmed.1001581)
Supplement: Table S5 — IPV and associated factors. (DOCX) [file pmed.1001581.s005.docx]

**Table S5: Domestic Violence and Termination of Pregnancy: Associated Factors**

| Association Sought | Paper | Outcomes | | CASP Score | Quality |
| --- | --- | --- | --- | --- | --- |
| **DEMOGRAPHIC FACTORS** | | | | |  |
| Age | Alio et al, 2011^75^ | | Compared to women over the age of 25 years, younger women are at greater risk of TOP if IPV is present. | 27 | High |
|  | Bourassa et al, 2007^30^ | | Women aged less than 20 years are more likely to report lifetime history of violence. Age is not significantly associated with past year physical or sexual violence. | 25 | High |
|  | Romito et al, 2009^29^ | | Women aged over 30 years at increased TOP risk if experiencing mainly psychological violence. | 25 | High |
|  | Roth et al, 2011^41^ | | No significant association found. | 24 | Medium |
|  | Wu et al, 2005^88^ | | No significant association found. | 23 | Medium |
|  | Helweg-Larsen et al, 2003^32^ | | Women aged 20-29 years experiencing IPV more likely to have TOP at any stage during follow-up than non-IPV counterparts. | 22 | Medium |
|  | Wiebe et al, 2001^57^ | | No significant association found. | 16 | Low |
| Marital status | Antai et al, 2012^73^ | | Single women are more likely than married women to report a history of IPV and TOP. | 26 | High |
|  | Bourassa et al, 2007^30^ | | Single women more likely to report IPV at TOP clinic. | 25 | High |
|  | Roth et al, 2011^41^ | | More likely to be single, divorced, separated or widowed than married. | 24 | Medium |
|  | Leung et al, 2005^87^ | | Women with a history of IPV are more likely to be single or separated. | 23 | Medium |
|  | Wu et al, 2005^88^ | | No significant association found. | 23 | Medium |
|  | Saftlas et al, 2010^43^ | | Women stating that they are ‘not in a relationship’ are at higher risk of IPV. | 22 | Medium |
|  | Glander et al, 1998^59^ | | Women seeking TOP who were abused are less likely to be cohabiting | 21 | Medium |
|  | Taft et al, 2004^91^ | | Women reporting IPV are more likely to be single, separated, divorced or widowed than married. | 20 | Medium |
|  | Evins et al, 1996^60^ | | Women seeking TOP are more likely to be single than married, divorced or separated. | 16 | Low |
| Race | Woo et al, 2005^54^ | | No significant association found. | 26 | High |
|  | Glander et al, 1998^59^ | | White women more likely to report IPV than women of other races. | 21 | Medium |
|  | Keeling et al, 2004^69^ | | No significant association found. | 21 | Medium |
| Ethnicity | Woo et al, 2005^54^ | | Women reporting positive IPV history are more likely to be Hispanic (Study based in Texas). | 26 | High |
|  | Wiebe et al, 2001^57^ | | No significant association found. | 16 | Medium |
| Education | Woo et al, 2005^54^ | | No significant association found. | 26 | High |
|  | Roth et al, 2011^41^ | | No significant association found. | 24 | Medium |
|  | Wu et al, 2005^88^ | | No significant association found. | 23 | Medium |
| Woman’s income | Roth et al, 2011^41^ | | No significant association found. | 24 | Medium |
| Household income | Woo et al, 2005^54^ | | Women reporting IPV are more likely to have low household income. | 26 | High |
| Woman’s drug and alcohol use | Leung et al, 2005^87^ | | Women reporting IPV are more likely to smoke/drink. | 23 | Medium |
|  | Wu et al, 2005^88^ | | No significant association found. | 23 | Medium |
|  | Glander et al, 1998^59^ | | Drug and/or alcohol involvement was found to be a reason for terminating a pregnancy in five women with a history of IPV, but none without. | 21 | Medium |
| Employment status | Roth et al, 2011^41^ | | No significant association found. | 24 | Medium |
|  | Gee et al, 2009^28^ | | Women out of work are at increased IPV risk. | 23 | Medium |
|  | Leung et al, 2005^87^ | | Women with a history of IPV are more likely to be non-skilled manual workers or unemployed. | 23 | Medium |
|  | Wu et al, 2005^88^ | | Type of occupation is not significantly associated with increased levels of IPV and TOP . | 23 | Medium |
|  | Keeling et al, 2004^69^ | | Not significantly associated with IPV and TOP. | 21 | Medium |
| Relationship problems/ discord | Bourassa et al, 2007^30^ | | Women who state their relationship is in difficulty or breaking down are also more likely to report IPV. | 25 | High |
|  | Glander et al, 1998^59^ | | ‘Relationship problems’ more likely to be primary reason for TOP in women with positive IPV history than those without. | 21 | Medium |
|  | Ely and Otis, 2011^38^ | | Women reporting physical IPV are more likely to report partner relationship problems and sexual discord. | 17 | Low |
| Living arrangement | Gee et al, 2009^28^ | | Women living alone or with someone other than their parents, boyfriend or husband are more likely to report IPV. Not shown to be statistically significant. | 23 | Medium |
| **REPRODUCTIVE HISTORY** | | | | |  |
| Parity | Roth et al, 2011^41^ | | No significant association found. | 24 | Medium |
| Gravidity | Roth et al, 2011^41^ | | No significant association found. | 24 | Medium |
| Gestation of termination of pregnancy | Woo et al, 2005^54^ | | No significant association found. | 26 | High |
|  | Jones et al, 2013^36^ | | 13.7% of women undergoing TOP at 12/40 report IPV; at 16/40 this has risen to 39.1%. | 24 | Medium |
|  | Roth et al, 2011^41^ | | Women reporting IPV more likely to be re-dated by ultrasound. | 24 | Medium |
|  | Kalyanwala et al, 2010^84^ | | Women reporting pregnancy as a result of forced sex more likely to have second trimester TOP. | 19 | Low |
|  | Wiebe et al, 2001^57^ | | No significant association found. | 16 | Low |
| Previous obstetric history | Roth et al, 2011^41^ | | Women reporting IPV more likely to have a history of miscarriage. | 24 | Medium |
| Number of previous termination | Fisher et al, 2005^51^ | | Women reporting three or more TOPs are 2.5 times more likely to have IPV history than those who do not. | 24 | Medium |
|  | Roth et al, 2011^41^ | | IPV more likely if previous TOPs reported. | 24 | Medium |
|  | Stenson et al, 2001^27^ | | Women reporting IPV are likely to have undergone more TOPs than those not reporting IPV. | 24 | Medium |
|  | Gee et al, 2009^28^ | | As the number of previous TOPs increases, the likelihood of IPV increases (univariate analysis only). | 23 | Medium |
|  | Laanpere et al, 2013^65^ | | Among 360 women reporting one TOP, 58 report IPV; at two TOPs, 41/213; three TOPs 51/181. | 23 | Medium |
|  | Silverman et al, 2010^44^ | | Male involvement in more than three TOPs increases the likelihood of perpetration of IPV. | 21 | Medium |
|  | Steinberg et al, 2011^42^ | | Women reporting previous TOPs are more likely to also report domestic violence (p<0.05). | 19 | Low |
|  | Ely and Otis, 2011^38^ | | Women reporting IPV are more likely to have a history of previous TOPs. | 17 | Low |
|  | Hedin et al, 2000^71^ | | Abused women reporting previous TOP: 2/23; non-abused women reporting previous TOP 11/184. | 17 | Low |
|  | Evins et al, 1996^60^ | | Abused women reporting previous TOP: 6/16; Non-abused women reporting previous TOP: 10/34. | 16 | Low |
| Sexual assault/rape | Holmes et al, 1996^61^ | | 50% rape related pregnancies end in TOP, with 29.4% rapes perpetrated by boyfriend and 17% by husband. | 21 | Medium |
| Contraceptive Use | Gee et al, 2009^28^ | | Women with a history of IPV are more likely to skip/go without/be unable to afford contraceptives. They are also at increased risk of using emergency contraceptives. | 23 | Medium |
|  | Ely and Otis, 2011^38^ | | Women with a history of IPV are less likely to use contraceptive. More likely to report that partner refuses to use condom. | 17 | Low |
|  | Hathaway et al, 2005^52^ | | Reports of birth control sabotage by male partner. | 13 | Very low |
|  | Thiel de Bocanegra et al, 2010^45^ | | Only women with a history of IPV studied: low overall contraceptive use within group irrespective of TOP history. Reports of both birth control sabotage by male partner and concealed use of birth control by female partner. | 13 | Very low |
| Pregnancy intention | Bourassa et al, 2007^30^ | | Women who choose to terminate a planned pregnancy are likely to report IPV. | 25 | High |
|  | Romito et al, 2009^29^ | | Women subjected to partner violence, especially physical or sexual, are more likely to state that their pregnancy was imposed upon them by their partner. | 25 | High |
|  | Raj et al, 2005^53^ | | Women in abused population more likely to report an unwanted pregnancy. | 22 | Medium |
| Partner knowledge of termination of pregnancy | Woo et al, 2005^54^ | | 17.2% respondents did not disclose pregnancy to partner. Recent physical abuse was twice as common among this group as disclosers. | 26 | High |
|  | Jones et al, 2011^39^ | | Partner less likely to know about decision for TOP if violent. | 22 | Medium |
|  | Glander et al, 1998^59^ | | Partner less likely to know about decision for TOP if violent (p=0.002). | 21 | Medium |
|  | Ely and Otis, 2011^38^ | | Women who report IPV are less likely to tell their partner about their decision to terminate. | 17 | Low |
| Partner support for termination | Jones et al, 2011^39^ | | Women reporting physical abuse or forced sex are more likely to report that their partner is not supportive of the TOP. | 22 | Medium |
|  | Glander et al, 1998^59^ | | Partner significantly less likely to support the decision to terminate if domestic violence is present (p<0.001). | 21 | Medium |
|  | Ely and Otis, 2011^38^ | | Abused women reporting that their partner supports TOP: 15/23.  Non-abused women reporting that their partner supports TOP: 116/149 | 17 | Low |
| Financing of termination | Ely and Otis, 2011^38^ | | Women with a IPV history are less likely to have their partner fund their TOP. | 17 | Low |
| Coercion into having termination | Wu et al, 2005^88^ | | 2.1% of participants stated they were being forced by their intimate partner into having a TOP. | 23 | Medium |
|  | Hathaway et al, 2005^52^ | | Some participants report feeling forced into having TOP (18%) or undergoing forced TOP (5%). | 13 | Very low |
|  | Thiel de Bocanegra et al, 2010^45^ | | Women reported pressure from partner to have TOP. One participant (n=53) reported being forced to attend a TOP clinic, but not actually undergoing procedure. | 13 | Very low |
| Birth control sabotage | Gee et al, 2009^28^ | | 16.7% women in violent relationships stated that their reason for not using birth control was that their ‘partner didn’t feel like it or wanted to get [her] pregnant’. | 23 | Medium |
|  | Hathaway et al, 2005^52^ | | Women report partner refusal to use birth control (condoms or withdrawal method), refusal to allow woman to use birth control (pill or tubal ligation), and birth control deception (lying about sterility or tearing condoms). | 13 | Very low |
|  | Thiel de Bocanegra et al, 2010^45^ | | 40% participants stated that they had been told by their partner not to use birth control; 19% had been prevented from obtaining birth control; 21% concealed their use of birth control. | 13 | Very low |
| Fetal sex | Puri et al, 2011^94^ | | Women report neglect, violence and feeling pressurised to have sex-selective TOP in cases of female fetus. | 14 | Medium* |
| **MEDICAL HISTORY** | | | | |  |
| Medical history | Leung et al, 2005^87^ | The presence of IPV lowers the physical, emotional, environmental and psychological quality of life scores of victims. | | 23 | Medium |
| History of child/other non-IPV abuse | Evins et al, 1996^60^ | 54.4% women reporting IPV reported that their perpetrating partner had a history of abuse as a child. | | 16 | Low |
| **IPV ENQUIRY AND INTERVENTION** | | | | |  |
| IPV Enquiry | Leung et al, 2002^33^ | 18.8% participants reporting recent abuse wished to tell gynaecologist/social worker about IPV with regard to further management/intervention.  Women in violent relationships not more likely to default from follow-up than women not in violent relationships. | | 24 | Medium |
|  | Roth et al, 2011^41^ | Women who did not answer IPV questions were more likely to: be Hispanic; have a history of a TOP; have earlier gestational age; or be at one particular site involved . | | 24 | Medium |
|  | Whitehead et al, 2005^90^ | 98% women felt comfortable filling out IPV questionnaire. | | 18 | Low |
|  | Evins et al, 1996^60^ | 80% women with IPV and 66.7% women without IPV knew of community IPV resources.  4% cite doctor as main source of information. | | 16 | Low |
|  | Wiebe et al, 2001^57^ | 50.9% women asked about IPV during time of universal screening. White women more likely to be asked than women of any other ethnic/racial group. | | 16 | Low |

*Quality scored using qualitative CASP form (marked /20)
