# Supplementary material for: Associations between Intimate Partner Violence and Termination of Pregnancy: A Systematic Review and Meta-Analysis
Source: PLoS Med. 2014 Jan 7;11(1):e1001581. doi: 10.1371/journal.pmed.1001581 (PMC3883805; doi:10.1371/journal.pmed.1001581)
Supplement: Table S6 — Lifetime prevalence of intimate partner violence in women presenting for termination of pregnancy: meta-analysis regression to compare odds ratios between study categories. (DOCX) [file pmed.1001581.s006.docx]

**Table S6: Lifetime prevalence of intimate partner violence in women presenting for TOP: Meta-analysis regression to compare odds ratios between study categories.**

| Predictor | Study categories | Odds Ratio | 95% CI |
| --- | --- | --- | --- |
| GNP per head | ≥$10,000 | (reference) |  |
|  | <$10,000 | 0.77 | 0.30 to 1.94 |
|  |  |  |  |
| GNP per head | ≥$10,000 | (reference) |  |
|  | <$10,000 (excluding China) | 0.89 | 0.22 to 3.57 |
|  | China | 0.69 | 0.22 to 2.23 |
|  |  |  |  |
| Study Design | RCT | (reference) |  |
|  | Case-Control | 1.38 | 0.44 to 4.31 |
|  | Cohort | 1.72 | 0.39 to 7.50 |
|  |  |  |  |
| CASP score | ≥ 25 | (reference) |  |
|  | 20-24 | 0.47 | 0.077 to 2.81 |
|  | 15-19 | 1.35 | 0.29 to 6.26 |
|  | <15 | 1.00 | 0.25 to 3.96 |
|  |  |  |  |
| Date of publication | Per year | 0.91 | 0.83 to 1.004 |
|  |  |  |  |
| Total sample size | ≥ 1,000 | (reference) |  |
|  | 100 to 999 | 2.77 | 0.87 to 8.83 |
|  | <100 | 1.17 | 0.42 to 3.26 |
|  |  |  |  |
