# Supplementary material for: Associations between Intimate Partner Violence and Termination of Pregnancy: A Systematic Review and Meta-Analysis
Source: PLoS Med. 2014 Jan 7;11(1):e1001581. doi: 10.1371/journal.pmed.1001581 (PMC3883805; doi:10.1371/journal.pmed.1001581)
Supplement: Text S1 — Protocol. (DOCX) [file pmed.1001581.s007.docx]

**Text S1: Protocol**

**Systematic review of associations between domestic violence and termination of pregnancy**

**Lead: Dr Susan Bewley, King’s College London**

**Study Protocol**

***Background:*** There is a high rate of domestic violence among women seeking termination of pregnancy. There are also associations with demographic factors and substance misuse.  Studies have different findings depending on definitions of violence and abuse (physical, sexual, emotional), methodology of studies and the populations investigated. There is also thought to be associations with various demographic and health factors such as age, substance abuse and history of termination.

***Primary Objectives:***

- To perform a systematic review and meta-analysis of the association between domestic violence and termination of pregnancy

***Secondary Objective:***

- To determine any additional factors associated with both domestic violence and termination of pregnancy

***Methods:***

Population: women who plan to undergo, or who have undergone, a termination of pregnancy

Exposure: domestic violence as reported by the woman or her partner

Study Inclusion Criteria:

- Includes women that were seeking or had undergone a termination of pregnancy and studied at least one aspect of domestic violence in this group;
- Randomised control trial, case-control study, cohort study, cross-sectional analysis, experimental study, or secondary study with data of interest;
- Peer reviewed.
- No restrictions on setting or time.
- Quantitative data not necessary for inclusion.

Study Exclusion Criteria: none.

Comparator group: as reported within each paper

Outcome: association reported between domestic violence and termination of pregnancy

***Search Strategy:*** A comprehensive literature review using the databases MEDLINE, Embase, PsycINFO and Maternity and Infant Care will be performed. Search strategies will be tailored to each database so as to employ the correct MeSH terms. Where possible, both MeSH and free text terms with synonyms will be used so as to increase identification of potentially relevant studies. Where MeSH terms are not used, free text only will be used. A separate search of Web of Science will be undertaken in order to capture any grey literature. Reference list review of included papers will be carried out.

No limit will be applied to the databases either in terms of publication date or language, with all databases searched from inception.

***Study Quality Assessment Checklist:*** Study will be assessed using the PRISMA guidelines and checklist. Study quality will be assessed using modified CASP scoring carried out by two authors independently.

***Data Extraction Strategy:*** Two authors will manually enter data into a standard data extraction form for each study and then synthesise into Tables.
